# The Interaction of the Gut Microbiota with the Mucus Barrier in Health and Disease in Human

**DOI:** 10.3390/microorganisms6030078

**Published:** 2018-08-02

**Authors:** Anthony P. Corfield

**Affiliations:** Mucin Research Group, School of Clinical Sciences, Bristol Royal Infirmary, Level 7, Marlborough Street, Bristol BS2 8HW, UK; mdapc@bristol.ac.uk

**Keywords:** gastrointestinal, glycoprotein, glycosylation, glycan, glycocode, microbiota, mucus, mucin, mucosal

## Abstract

Glycoproteins are major players in the mucus protective barrier in the gastrointestinal and other mucosal surfaces. In particular the mucus glycoproteins, or mucins, are responsible for the protective gel barrier. They are characterized by their high carbohydrate content, present in their variable number, tandem repeat domains. Throughout evolution the mucins have been maintained as integral components of the mucosal barrier, emphasizing their essential biological status. The glycosylation of the mucins is achieved through a series of biosynthetic pathways processes, which generate the wide range of glycans found in these molecules. Thus mucins are decorated with molecules having information in the form of a glycocode. The enteric microbiota interacts with the mucosal mucus barrier in a variety of ways in order to fulfill its many normal processes. How bacteria read the glycocode and link to normal and pathological processes is outlined in the review.

## 1. Introduction

The mucosal protective barrier is a feature of higher animals and has been developed and maintained throughout evolution [1,2]. The family of mucus glycoproteins, the mucins, are an integral part of this barrier and also feature throughout evolution [3,4]. A principal character of the mucins is their glycosylation, a high proportion of their molecular weight consists of carbohydrate in the form of oligosaccharides, or glycan chains [5,6,7,8]. The glycans are made up of a sequence of monosaccharides and are biosynthesized and degraded by enzymes that recognize the glycan structures and their linkages. The sequences generated and expressed are known and predictable, due to their mode of synthesis. They form a glycocode [9] where the sequence is recognized by proteins that play a role in mucosal protection, resident and pathogenic microorganisms, transient food borne bacteria interactions, and innate and adaptive immune responses [10]. This glycocode is species and tissue specific and is closely linked to the microbiota associated with individual mucosal surfaces [10,11,12]. The expression of the mucins in the mucosal defensive barrier is dynamic and is known to adapt to mucosal changes, in order to maintain optimal protection. A number of diseases have been identified which relate to aberrant glycosylation of the mucins and have been used as biomarkers for these pathological conditions [13,14,15]. The known diseases include genetic based abnormalities [16,17] in addition to tissue specific and environmentally effected changes which would influence mucins and lead to mucus which does not function effectively and results in reduced mucosal protection and the appearance of pathological features [7,18,19,20,21,22,23,24,25,26].

This review will identify the principal characteristics of the mucosal protective barrier in the gut, with regard to the role of the mucins and their glycosylation.

## 2. The Structure of the Mucus Barrier

The mucus barrier is the primary defensive layer at the surface of mucosal surfaces throughout the body of higher animals. It is a multi-component structure, which is integrated to ensure both protection and communication. This is achieved in several ways depending on the individual components.

The mucosal cells themselves are characteristic and many additional elements are derived directly from them [6,27,28,29,30]. The oral cavity and the oesophagus comprise squamous epithelial cell layers, while in the lower gastrointestinal (GI) tract a single layer of columnar epithelial cells are dominated by enterocytes. In addition, the intestine is well innervated and the enteric nervous system mediates gut motility, fluid exchange, blood flow, secretion, and barrier permeability through paracrine processes, while juxtacrine mechanisms occur via cell-cell contacts formed at the gap junctions. Both of these mechanisms are calcium dependent.

The adherent mucus is synthesized and secreted by the Goblet cells, located in all parts of the intestinal tract. Recent work has emphasized the range of Goblet cells found in the GI tract, together with specific functions relating to the mode of mucus secretion [28,29,30,31]. The function of the Goblet cells varies depending on their location in the small intestinal or colorectal crypts. The identification of a “sentinel” Goblet cell at the mouth of colonic crypts serves to underline the concept that Goblet cells vary depending on their intestinal location [32]. The number of vesicles found in these cells, together with the release of mucus into the crypt lumen is mediated to ensure channeled release and formation of mucus fibrils. This has recently been demonstrated in the lung for MUC5B [33,34] and is assumed to function in a similar manner in the gut with MUC2. The mucus product found at mucosal surfaces throughout the body is derived from the gel-forming mucins and is well recognized as part of the mucosal barrier with a characteristic thickness. The mucus thickness in the GI tract has been extensively analyzed and reported [35,36].

A contrasting viewpoint regarding mucus thickness has recently been reported, proposing that the mucosal contents govern the thickness of the mucus layer and this is region specific, occurring largely in the distal colon [37]. The observations in this case show no evidence for an adherent mucosal gel layer where fecal content is present. Instead mucus is attached to the fecal pellet and is absent from the surface of the epithelium. A functional role for the mucus, as proposed in the two-layer model, would therefore be redundant.

## 3. The Mucin Gene Family and Their Role in the Gut

The family of mucin genes currently includes 21 members. Their macromolecular structure is organized through disulfide bridges and some of the mucins also contain isopeptide linkages. They have been divided into two basic groups on the basis of their biological functions, secreted mucins and the membrane-associated mucins. The secreted members include the gel-forming mucins, important for mucus barrier formation at mucosal surfaces, and also secreted, non-gel forming mucins. The membrane-associated mucins are essentially components of the cell-surface glycocalyx and it is here that their glycans contribute to the carbohydrate rich surface involved in many interactions between cells and the external environment. Those mucins commonly found in the gut include the gel-forming mucins MUC2 (jejunum, ileum, and colon), MUC5AC (stomach), MUC5B (in submandibular and other salivary glands), MUC6 (stomach and ileum) and the non-gel forming mucin MUC7 (sublingual and submandibular glands). Membrane-associated mucins in the gut include MUC1, MUC3A/B, MUC4, MUC12, MUC13, MUC15, MUC17, MUC20, and MUC21. Each mucin has a typical protein domain structure which correlates with their secreted or membrane-associated nature. More information on the individual mucins can be found in the literature [6,8,38,39,40,41,42,43]. The major feature of all mucins is the proline-threonine-serine (PTS) rich domain, which contains the serine and threonine residues that form the glycosidic links to GalNAc, the first monosaccharide in the O-linked glycan chains, typical of mucins. The PTS domains are expressed as tandem repeats, thus generating a domain, which carries a large number of glycans. The size and pattern of these PTS domains varies between mucins. The main features of these molecules are shown in Table 1 and Table 2.

The mucins are essentially glycosylated polymer proteins, which have been evolved to function as part of the mucosal protective barrier and as cell membrane components presenting a characteristic glycoarray at the cell surface [5,6,39,44,45,46,47].

Studies on the evolution of both the mucins and protein glycosylation clearly demonstrate that these are biologically significant features. The origin of the mucins can be traced back to phyla associated with the early metazoan period [3,4,48], while the glycans show a similar evolutionary profile within the eukaryotes [1,49,50,51,52]. In contrast, the prokaryotes show a diverse range of protein glycans that vary from the eukaryotes in their structure and mode of metabolism [53]. This evolutionary data highlights the physiological consequences of mucin glycosylation and gives a perspective in relation to the current emphasis placed on DNA and protein sequence information.

## 4. Bacterial Species in the Human Gastrointestinal Tract

The GI microbiota shows characteristic patterns throughout the tract and this has implications for the nature of interactions between the bacterial cells and the mucosal surface glycoarrays. Oral cavity species include *Streptococcus*, *Prevotella*, *Porphyromonas*, and *Fusobacterium* strains [57,58], stomach accommodates *Streptococcus*, *Lactobacillus*, *Staphylococcus*, and *Peptostreptococcus* [59], while an abundance of more than 1000 species are found in the small intestine and colon [60,61]. These are largely anerobes, with 2–3 times more than facultative anaerobes and aerobes. The most common species are in the *Firmicutes* and *Bacteroidetes*, with fewer *Proteobacteria*, *Fusobacteria*, *Cyanobacteria*, *Verrucomicrobia*, and *Actinobacteria* strains. Ethnicity has also been shown to influence the GI tract microflora [62], this needs to be considered when comparisons between different population groups are made.

The ability of the human enteric microbiota to turn over mucus in the intestinal mucosa depends on the production of a series of hydrolytic enzymes, which degrade the mucus glycans to yield monosaccharides which serve as an energy source for the microbiota. The glycohydrolases adapted to the blood group of each individual and this has been demonstrated for mucin oligosaccharide degrader (MOD) strains [63,64].

Among other bacterial species that have special relevance for the mucins is the anaerobe *Bifidobacteria*, which are abundant in early life, especially in breast fed infants [65,66]. They are able to digest a range of host and diet derived glycans, including mucus and mucins. Evidence supporting this feature is the selective expression of carbohydrate transport systems and many proteins, which catalyze the degradation and metabolism of a variety of carbohydrates including low molecular weight oligosaccharides [65], polysaccharides such as glycogen, pullulan, starch, maltodextrin, and amylopectin [65] and mucins [67].

*Lactobacillus* species play a significant role in normal gut glycan metabolism and have been widely used as probiotics [68,69,70]. In addition, binding to intestinal mucus and mucins has been demonstrated [71,72]. A similar situation exists in the female reproductive tract, where the mucus layer in the vagina is normally colonized by *Lactobacillus* strains, and where reduction or loss of these species results in abnormal colonization, largely *Garderella* spp., and the development of bacterial vaginosis occurs and can be treated by probiotic *Lactobacillus* administration [73,74,75].

An important group of bacteria that have major roles in the metabolism of mucins in the gut are *Akkermansia* spp. [76,77]. Originally isolated from the gut flora in 2004 with mucin as a sole carbon source it was named after the Dutch microbiologist Antoon Akkermans [78]. Akkermansia spp. has been identified as human gut species present from early childhood [76,78,79,80]. In accord with its location in the mucus layer of the gut many strains have carbohydrate metabolic proteins in their genome and therefore are well able to metabolize and utilize mucus and its monosaccharides from the secreted gel-layer [76,81].

A fundamental trait of these bacteria is cross-feeding, whereby the carbohydrate metabolic capacity of individual species at any one location contributes to the energy requirements of all species present. This means that although some strains may not express all enzymes necessary for generation of monosaccharide substrates the total flora is able to achieve this and provide monosaccharides for all strains present [82,83,84].

Developmental aspects are important and age related variations are found throughout life [85,86,87,88]. The expression of mucin glycosylation during development has been followed in mammalian species and the fruit fly *Drosophila melanogaster*, widely used as a research organism [89]. In the fruit fly, detection of *O*-glycans showed limited and precise tissue patterns in embryonic tissues and larval imaginal disks [90]. In mammals similar developmental expression of *O*-glycans in organs and tissues has been detected [91] and the maintenance of UDP-GalNAc:polypeptide alpha-*N*-acetylgalactosaminyltransferases (ppGalNAcT’s) through evolution from *Drosophila* to mammals strongly suggests that *O*-glycans have been specifically selected and conserved for the biological roles linked to developmental events [92].

Patterns of intestinal mucin gene expression during different stages of fetal developments have been reported and reviewed [93,94], but give no indication of glycosylation arrays. Early histochemical studies of mucins in the human fetal intestine showed similar sialylation and sulphation patterns to adult colonic tissue [95,96,97]. However, a closer chemical examination of the *O*-glycans in fetal intestinal tissues showed relevant variations to the adult state. Although most of the *O*-glycan structures were the same as those found in adults, with variation of the linkage to the peptide through the different core structures shown in Table 3, largely core 2, and some core 1, 3 and 4 based structures, but no Sd^a^ glycans (Neu5Acα2-3(GalNAcβ1-4)Galβ1-3/4GlcNAcβ1-3GalNAc-R) were observed and the acidic gradient, due to sialylated and sulphated *O*-glycans was not detected. A constant pattern of *O*-glycans was found along the length of the intestine, in contrast to the variation as seen in the adult colon [98].

The question that arises from these results is whether the developmental regulation of intestinal *O*-glycans relates to the bacterial flora present. In the amnion and fetus, there is essentially no bacterial presence and normal colonization initiates at birth. This suggests that there is a programmed glycomic response to the introduction of bacteria to the gut and certain *O*-glycan structures, in particular the Sd^a^ antigen, and their location in the gut are relevant to the development and establishment of a stable and normal flora and an effective and dynamic mucus barrier.

At the early stages of life there is evidence that glycosylation plays an important role in establishing the stability and protection of the GI tract. This is apparent at birth, during lactation, through weaning, and the subsequent progression to adulthood. Much of the data derived for this concept has come from the dietary profile of children from birth onwards. It has been reported that the glycosylation of milk proteins varies during lactation, this has been shown for the major family of milk glycoproteins, the caseins in both man and cow [99,100] and also for human milk lactoferrin [101,102]. In keeping with this concept, the pattern of low molecular weight oligosaccharides present in mothers-milk is known to change during lactation [103,104]. The oligosaccharides are thought to play a role as prebiotics, as inhibitors of pathogenic or detrimental bacterial binding to the developing gut mucosa and in order to promote colonization of beneficial stains and to establish a normal flora [105,106,107,108,109].

With the completion of the lactation period and the change in the diet leading into weaning a series of changes is initiated which subsequently results in the establishment of the adult pattern of intestinal microbiota. This has been noted in human and animal studies [87,110,111,112]. The aging process has a profound influence on the composition and homeostasis of the human microbiota and also impacts on mucin glycosylation [113,114] and host immune system [115,116]. A reduction of the salivary mucins MUC5B and MUC7 was found [113] and a reduction in the diversity of the microbiota was observed [117,118]; a decrease in A. muciniphilia has also been reported [76]. In contrast, a greater array of species was detected in another study [119].

The diet has been identified as a strategic factor maintaining the flora [120]. Many of the diseases associated with advanced age also correlate with changes in the gut microbiota, mucus expression, and glycosylation [121]. In elderly patients with *Clostridium difficile*, a lower microbial diversity was found [122], while a wider variety of micobiota was found in aged IBD patients. *H. pylori* infection was found to correlate with histological and serological changes in the elderly [123]. Specific probiotics have been adopted to stabilize and maintain the microbiota in older individuals [124].

## 5. Mucin Glycosylation and the Sugar Code

### 5.1. Bulk Properties—Gel Formation and Viscoelasticity

Before considering the sequence of the mucin glycans it is necessary to address the primary physical properties of the mucins in vivo. These are the characteristics that contribute to the barrier function of the secreted mucus and are evident in the mucus layers found in the GI tract. The secreted mucins form viscoelastic gels through generation of molecular networks. The gel forming mucins display rheological properties through bulk mucus flow. They are both viscous and elastic, fundamental properties due to covalent and reversible interactions, mediated by the concentration of the gel forming mucins themselves, environmental salt concentration, and local pH [125]. Mucin rheology should be regarded as a fundamental physiological property of mucins reflecting selective molecular design throughout evolution [126,127,128]. Recently the biological importance of the GI mucus barrier as a two-layer system, initially described by the Allen group [35,129], has been demonstrated to comprise an inner, adherent gel on the surface of the mucosa, which is devoid of enteric bacteria, and an outer, thicker layer, that is constantly being degraded and shed, but which harbors a bacterial population [130,131,132,133].

The mucus barrier is dynamic. In order to maintain its primary functions in mucosal protection it is continuously renewed at a rate sufficient to balance the normal destructive forces leading to the constant erosion and loss of the outer layer.

### 5.2. Mucin Glycans; Sequence, Topography and Mucosal Interactions 

The glycosylation of mucins is a selective process and derives from the biological design to yield a high molecular weight polymer than can be secreted and will form a gel or has a recognition function and forms a part of a glycoarray at the surface of the in the glycocalyx. The formation of viscoelastic, secreted polymers can be achieved without the range of glycan structures found in the gel-forming secreted mucins. This suggests that the selection of mucin glycosylation is designed to provide recognition information in addition to the physicochemical properties.

The carbohydrates are well suited, both chemically and physiologically, to generate a broad variety of glycan structures that have sequential identity and therefore information [134,135]. Unlike the nucleic acids and proteins, which have linear structures only, the glycans can form branched structures in addition to linear chains. The basic building blocks the monosaccharides are epimers of each other and exist as α- or β-glycosides. Thus the anomeric configuration, regiochemistry, and stereochemistry of the glycosidic linkage are basic features of glycan chains [136]. Protein glycosylation appears in number of well-known forms and which are outlined in Table 4.

The mucins are well-known as proteins carrying a wide range of glycans of the “mucin type”. This abundance of glycans takes the form of *O*-linked glycans attached to serine and threonine (ser/thr) groups in the mucin polypeptide tandem repeat, PTS rich domains. Recent analysis has implicated the link to either serine or threonine as a selective process with biological significance. Comparison of serine-linked versus threonine-linked mucin *O*-glycans shows different properties in their interaction with lectins, implying a potential for different functions based on the type of *O*-glycan linkage. [137]. The linkage sugar is *N*-Acetyl-d-galactosamine (GalNAc) and, as noted below, the transfer of this initial sugar is catalyzed by a family of *N*-acetyl-d-Galactosaminyltransferases, which show specificity with regard to the mucin peptide sequence, including the proximity of other ser/thr attachment sites and whether they are already substituted by a GalNAc residue. The chemical and biochemical complexity of this glycosylation step emphasizes the biological importance of this initial event and coordinates the mucin for its physiological role at its site of biosynthesis and secretion [138,139,140].

Extension of the initial GalNAc generates a series of mucin core structures. Eight core structures have been identified, of which only four show widespread abundance. These are shown in Table 3.

**Figure 1:**
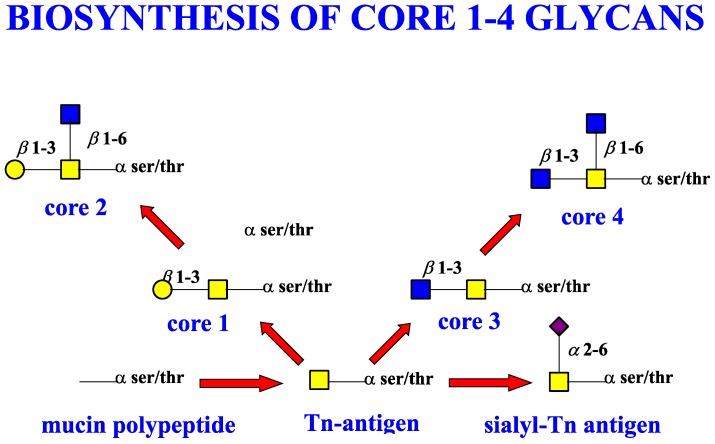
Biosynthetic Pathways leading to Mucin Core 1–4 Structures. Abbreviations and monosaccharide symbols are given at the end of the paper.

The structure of cores 2 and 4 demonstrates the potential for the formation of branched structures, in contrast to the nucleic acids and proteins. The branching option expands the viable range of *O*-glycan structures and correlates well with the extensive scope of glycans carried by mucins. The core structures may remain as short oligosaccharides, but the majority are extended. Larger glycan structures are achieved through the action of a range of well-established pathways as shown in Figure 1.

The extension process enables the formation of larger and more branched glycans. Some of the peripheral glycan structures are shown in Table 5. The scope for formation of these glycans in the mucins includes transfer of *L*-fucose, *N*-acetylneuraminic acids (sialic acids), acetylation, sulphation, and methylation [5,20,49,141,142,143,144].

*N*-glycosylation is also a significant feature of mucin glycosylation, but fewer *N*-glycan chains are found compared with the *O*-glycans. They occur principally in the membrane-associated mucins, but show discrete patterns in MUC1, MUC4, and MUC16. MUC1 contains *N*-glycans in both the PTS and SEA domains, while in MUC4 these are only found in the EGF domain and MUC16 expresses *N*-glycans in its PTS region [40].

The location of *N*-glycans on mucin peptide and other glycoproteins is determined by recognition of a tripeptide sequence, asparagine-X-serine, where X is any other amino-acid, except proline. Considerable structural variation of the *N*-glycans occurs in nature, and this range of glycans is derived from three main core forms, as shown in Figure 1. The cores are extended to create the series of *N*-glycans found in nature and accordingly in the mucins. Different numbers of antennae are known, bisecting GlcNAc is also present in certain cases and an internal fucose, attached to the GlcNAc linked to asparagine also occurs. Oligo-mannose forms, and complex forms terminated with a sialyl-*N*-acetyl-lactosamine trisaccharide and hybrid forms are common (see Figure 1). As with the *O*-glycans, noted above, the *N*-glycans possess a variety of different peripheral substitutions, leading to the profusion of *N*-glycans that have been detected and reported in glycoproteins. The *N*-glycans play important roles in mucin peptide processing, which occurs during biosynthesis [6,145,146,147].

An unusual type of glycosylation involving a single alpha-mannose unit attached through a C–C (carbon–carbon) linkage to peptide tryptophan residues located in mucin peptide WXXW motifs has been reported [148]. This novel form of glycosylation has been identified in the CysD domains of the secreted mucins, MUC2 (2 units), MUC5AC (9 units), and MUC5B (7 units). It has been proposed that these units function in protein folding, subcellular localization and trafficking [149,150]. *C*-mannosylation in MUC2, MUC5AC, and MUC5B is required for maturation and secretion. Deficient *C*-mannosylation of mucins results in their inability to exit the Endoplasmic Reticulum (ER) and leads to ER stress [43].

An important feature of glycosylation is its tissue and cell specificity. As stated above there are many glycan structures associated with mucins and it is clear that that same mucins are expressed in different organs, tissues, and cells. A good example of this is MUC5AC, which is expressed in the respiratory tract [151], stomach [152,153], gallbladder [154], conjunctiva and tear film [155,156], middle ear [157,158], prostate [159], and the female reproductive tract [160,161].

The need to provide optimal protection at different mucosal surfaces imposes a design and synthetic requirement for mucin glycosylation. The defensive processes necessary will depend on the mucosal surface in question and this fits well with the opportunity to biosynthesize mucin glycan sequences, which are adapted to the needs of each mucosal surface. It is known that the glycobiome, which has the ability to glycosylate individual proteins to yield distinct and discrete glycoforms, will have ideal function at their site of synthesis [5]. A good example is the glycosylation of MUC2 in the human GI tract. Regional patterns of MUC2 glycosylation occur from the small intestine through to the rectum, largely through sialylation and glycosulphation [162,163]. These patterns were constant when examined in more than 50 normal individuals [164] and in patients with ulcerative colitis, where aberrant mucin glycosylation is associated with the disease, recovery is accompanied with a return to the normal healthy glycosylation profile. In addition to the GI tract, characteristic mucin glycosylation profiles have been found in the oral cavity [165], the pancreas [166], the ocular surface and conjunctiva [155,167], the respiratory tract [168], human sperm [169], and the female reproductive tract [170].

It is clear that the variety of glycans found in mucins is a molecular design feature adopted and optimized throughout evolution. The glycocode is therefore well suited to the biological requirements of mucins as mucosal barrier components displaying dynamic, sequence based information.

As well as the wide ranging patterns of glycosylation found in mammals and especially in man, there are a number of human features which indicate that the sugar code is an integral part of our normal existence giving us unique labels at an individual level and establishing molecular recognition which govern interactions with our environments. The human blood group system is well known to rely on glycan sequences for its recognition [171,172,173]. The human blood groups found on proteins include the ABO(H) antigens, the Lewis antigens [174], the Sd^a^ antigen [175,176], and the i and I blood groups [177]. Much of the immunochemistry was established through the work of Karl Landsteiner [178], Elvin Kabat [179], Walter Morgan, and Winifred Watkins [172]. The development and conservation of the human blood group system has been confirmed through evolutionary study [180] and serves to emphasize the biological relevance and magnitude of this recognition system. These structures are carried on glycan chains of type 1 (Galβ1-3GlcNAc), common in *O*-glycans, or type 2 (Galβ1-4GlcNAc), mostly found in *N*-glycans and type 3 (β1*-*3 GalNAc-αser/thr-) associated with mucins. Some of the key glycan structures are shown in Table 5.

The blood group antigens are essentially expressed on the surface membranes of erythrocytes red cells. However, the expression of the same structures on mucosal glycoproteins is regulated through the glycosyltransferase FUT2, also known as the secretor gene. Individuals who have this fucosyltransferase transfer fucose to glycoproteins to give a α1-2 linkage [181,182]. These individuals are able to express blood group antigens on mucosal surfaces and are termed secretors. In contrast, those who have no FUT2 gene do not show the blood group antigens on their cellular glycoproteins and are known as non-secretors [181,183]. In addition to the molecular identification specified by the FUT2 gene it is also directly related to disease and is known to mediate infection and susceptibility [183,184]. The ABO blood group antigens expressed on erythrocytes (red cells) have been shown to modulate the pattern and arrangement of sialylated glycans on the erythrocyte surface [185]. A further feature of blood group activity and secretor status has been demonstrated for salivary MUC5B. The non-secretors had a higher sialylated form of MUC5B, with increased sialyl-Lewis^a^, compared with the secretors [186]. Thus demonstrating that mucin glycosylation depends on both blood group and secretor status.

**Figure 2:**
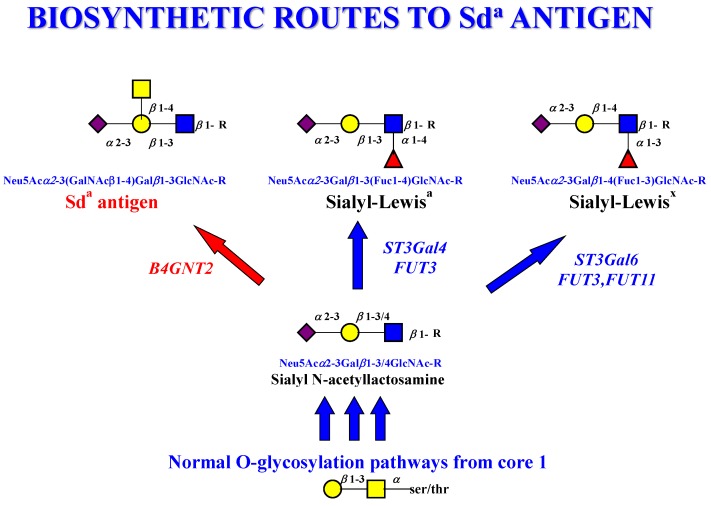
Biosynthetic Routes to the Sd^a^ antigen. The sequential steps leading to the Sd^a^ antigen from core 1, via sialyl-*N*-acetyllactosamineare shown. The individual glycosyltransferases for each step are indicated. The red arrow indicates the major pathway, while the blue arrows indicate competing steps to the sialyl-Lewis^a^ and sialyl-Lewis^x^ antigens. Abbreviations and monosaccharide symbols are given at the end of the paper.

The Sd^a^ antigen is commonly found in the normal colon [175] and its formation is regulated by the addition of β1-4GalNAc by the B4GALNT2 glycosyltransferase [187,188]. This contrasts with the Lewis and sialyl-Lewis antigens, which are normally only found at low levels. The biosynthetic pathway leading to the Sd^a^ antigen includes the intermediate structure sialyl-*N*-acetyllactosamine (Neu5Acα2-3Galβ1-3/4GlcNAcβ1-) and this represents an important branch point in the pathway as it may be converted to the Sd^a^ antigen, sialyl-Lewis^a^, or sialyl-Lewis^x^, as shown in Figure 2.

**Figure 3:**
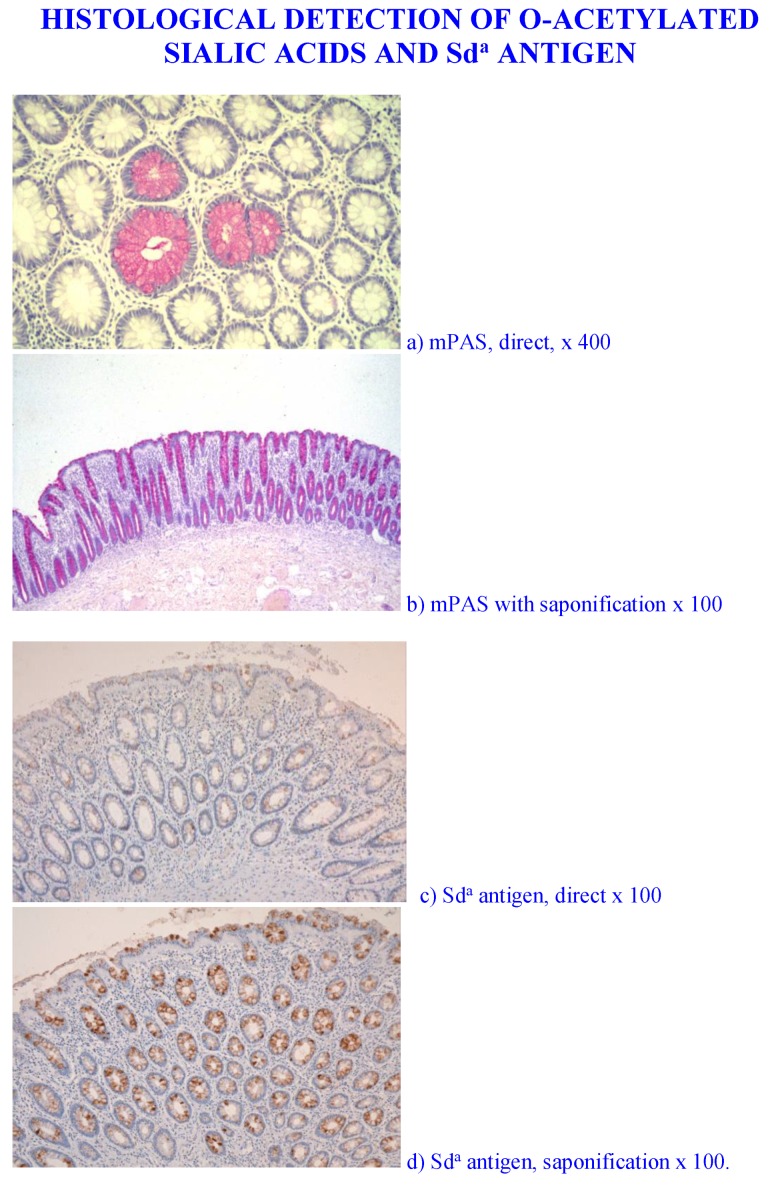
Histological Detection of *O*-acetylated Sialic acids and Sd^a^ antigen. The *O*-acetylated sialic acids detected by the mPAS stain, directly (**a**) and with saponification, (**b**) this shows a longitudinal section of the mucosa, in contrast to a, c, and d. Also note the difference in magnification. Direct staining for the Sd^a^ antigen with the KM694 antibody (**c**), and with saponification (**d**) is shown.

As demonstrated by chemical analysis on normal colonic mucus Sd^a^ antigen is the major structure found [162,163]. Unpublished data from our laboratory has demonstrated that the normal colonic mucus Sd^a^ antigen sialic acids are *O*-acetylated. Figure 3 shows the Sd^a^ antigen, detected with the KM694 antibody is sensitive to saponification with mild alkali [189,190,191] and adds a further regulatory asset to the antigen as the *O*-acetylated sialic acid is resistant to sialidase action.

*O*-Acetylation of sialic acids is well known to be a major modification in human colonic mucus. The demonstration of individual glycoproteins as carriers of *O*-acetylated sialic acids has not been widely studied. The human colonic mucins are a major carrier of *O*-acetylated sialic acids. The Sd^a^ antigen is one of many sialylated glycans carried by the mucins and is a focus of attention in this review.

It is also known that a small proportion of the general population do not express *O*-acetyl sialic acids and are known as sialic acid non-*O*-acetylators. These individuals can be detected using the mPAS (mild periodic acid/Schiff) stain with and without prior saponification. The biological adaptation to the absence of these sialic acids has not been examined. There is no indication whether they are more susceptible to gastrointestinal disease, or whether a natural adaptation occurs, as in the case of blood group secretors and non-secretors [186], with a corresponding glycobiological modification.

## 6. Mucin Glycans as Biological Arrays Linked to Function

The mucins represent the presentation of an array of glycan structures at whichever site they are expressed, as noted above. At many mucosal surfaces this mucin glycoarray interacts with the bacterial flora present under normal conditions. Much recent work in this area has identified microbiota which interact with different mucosal surfaces and which are adapted to each specific mucosa. The human gut has been widely examined [12,192,193,194,195,196,197,198].

The mucins are designed to provide defense at mucosal surfaces in many different ways and this reflects the adaptability of these molecules for this function. The basic organization of mucin protein domain composition and their glycosylation allows adaptation to the demands posed at each mucosal surface. As the production of the mucins is dynamic it is ideally adapted to respond to developmental and environmental changes that are expected. In the GI tract this is apparent at birth, during lactation, weaning, and in adulthood.

As mentioned above the mucosal barrier in the GI tract shows a mucus gel layer of differing thickness, depending on the location in the tract. The stomach and colon have a gel layer of about 700 µm, while the small intestinal thickness ranger between 150 and 300 µm [6,35,36]. The colonic barrier consists of two secreted mucus layers; these are essentially composed of MUC2. A major feature of these two layers is the distribution of bacterial populations. The inner, adherent secreted mucus is free of microbes, while the outer layer is colonized by the enteric gut bacterial flora. The sophistication of this system is apparent with the identification of different types of Goblet cells, which synthesize and secrete the mucus along the crypt in the human colon. Indeed a “sentinel” Goblet cell has been identified, positioned at the top of each colonic crypt. Endocytosis of TLR generates MUC2 secretion, together with an intercellular gap junction signal, which induces MUC2 secretion in adjacent Goblet cells and thus regulates the entry of bacteria into the crypt [28,32].

In contrast to the continuous, two-layer system, a recent report has presented data showing that the luminal contents of the distal colon have an influence on the location of mucus [37]. The report shows that mucus covers the feces, but not the distal colonic epithelium. As a result it confines the enteric microbiota to the surface of the feces and prevents it remaining in the vacant distal colon. Further work is required to confirm or refute this observation and it underlines the importance of regular review and interpretation of existing data.

The apical glycocalyx is ubiquitous to all cell types and is essential for normal cell interaction with neighboring cells and the external environment. It provides a platform for communication and links with signaling pathways within the cells. In common with the secreted mucins it has a characteristic composition at each mucosal surface. The membrane-associated monomeric mucins form a significant proportion of the molecular makeup of the glycocalyx and accordingly create a cell surface anchored glycoarray. Typically MUC1, MUC4, MUC12, MUC16, and MUC20 are found, with MUC1 present in most mucosal surface membranes [5,6,40,45,46,199,200,201,202].

## 7. Screening for Mucin Glycans and Mucin Glycan Engineering

The progress made in understanding mucin structure, organization, synthesis and degradation relied on improvements in technology. In addition, access to glycomic based databases has provided a reliable and constantly growing source of information for structural and functional aspects [203]. The two most widely used databases are CaZy, the Carbohydrate-Active enZYmes (CaZy) Database (http://www.cazy.org), and The Consortium for Functional Glycomics (http://functionalglycomics.org). A consequence of the increased biological interest in glycans has been a focus on the chemistry–glycobiology frontier and the need to understand chemical and physical aspects of all glycans [204].

The detection, isolation, and characterization of glycans has been improved through the production of reagents together with chemical, biophysical and biochemical methodology [205]. The techniques best suited to and most widely used in glycan isolation, detection, and assessment are Affinity Chromatography, which employs an immobilized binding protein on a suitable support such as Affi-Gel or Sepharose [206,207,208]. This can be used simply to bind the target glycan and separate it from all other compounds in a tissue or cell preparation. It can also be used to calculate the strength of binding and generate a k_d_ value when compared with a glycan not bound by the protein. The strength of binding can also be calculated using isothermal titration calorimetry [209,210]. The change in enthalpy is measured, in a microcalorimeter, for varying concentrations of the glycan at constant glycan binding protein concentration, and used to calculate the k_d_ value. Surface plasmon resonance has also been widely adopted to follow the kinetics of reaction and relies on the reflection of polarized light as the glycan is allowed to flow over the immobilized glycan binding protein [211,212]. Again values for the k_d_ of the reaction can be obtained. Fluorescence polarization techniques also allow measurement of k_d_ values. Characterization of non-covalent interactions between glycans and specific proteins can be measured using mass spectrometric and NMR methods.

The profusion of techniques existing for detection of glycans has led to the design of strategies for analysis of glycosylation patterns. The classical methodology for optimal glycan structural analysis is mass spectroscopy or NMR if sufficient probe is available, usually after HPLC separation of released glycans [213,214,215,216,217,218].

The recognition of specific glycan sequences can be monitored using proteins that bind to such glycans. Many of these have been used to probe for the presence and cellular and subcellular location of glycan motifs in tissues and cell lines, which express mucins.

The majority of these proteins are lectins or adhesins, isolated from microbial, plant and animal sources, readily available commercially and used widely as standard reagents [219,220,221,222,223,224,225,226]. There is a large literature on this topic and it is not the main focus for this review, however a brief overview with a small selection of references serves to indicate the important links in relation to mucin glycosylation and its biological recognition. An overview of current knowledge can be found in Essentials of Glycobiology, Third Edition [227].

R-type lectins are a superfamily of proteins, which contain a carbohydrate binding module (CBM, see below), and which bind to β-galactose or *N*-acetylgalactosamine. This is a large family and includes the GalNAc transferases involved in mucin synthesis, mannose receptors, bacterial lectins, invertebrate lectins, bacterial hydrolases, plant toxins, and *Drosophila* lectin [228].

L-type lectins are derived from leguminous plants, with glycan binding proteins from other eukaryotic organisms; they bind to a range of different glycans. Concanavalin A binds to glucose and mannose, while *Sambucus nigra* and *Maackia amurensis* lectins show affinity for sialylated oligosaccharides [229].

The P-type lectins recognize mannose-6-phosphate (M6P) carried on *N*-glycans. Glycoproteins that carry the M6P motif are generated through a series of steps and are delivered to the lysosomes. M6P acts as a translocation signal for lysosomal proteins.

C-type lectins are the largest and most diverse family. They are calcium dependent, with homology in their CBMs and include the collectins, selectins, endocytic receptors, and proteoglycans and may be either secreted or membrane bound. Fundamental conserved determinants implicated in glycan binding are the EPN motif promoting Man, Glc, Fuc, and GlcNAc recognition and the WND motif for Gal and GalNAc [230].

I-type lectins have binding domains which have homology with the immunoglobulin superfamily. They include the selectin family, which bind α2-3, α2-6, and α2-8 linked sialic acids. The specificity varies between the selectins and also includes Neu5Ac or Neu5Gc identification and *O*-acetylation patterns [231,232].

The galectins are typical β-galactose binding proteins found in vertebrate and invertebrate forms and sharing CBM homology. They exist as three major groups, (1) prototypical having only one CBM and which bind as homodimers, (2) chimera type, a single CBM with an attached proline rich peptide, and (3) tandem repeat which have two CBMs linked by a peptide [233,234].

Certain viral strains have also been used to screen for sialic acids and their *O*-acetylated forms [235,236,237,238]. Viral proteins that show hemagglutinin binding properties and those that have specific esterase activity for 4-*O*-acetylated sialic acids have been reported [238]. Recently a series of virolectins from nidovirus strains have been isolated and used to probe for *O*-acetylated sialic acids [239]. Dual function hemagglutinin-esterase envelope proteins were found to show very selective, differential binding patterns when used in soluble form. Discrimination between 4-O-Ac, 9-O-Ac, 7,9-diOAc, and 4,9-diOAc was possible and differential expression was revealed in human and mouse tissue arrays. This shows a pattern of sialic acid *O*-acetylation, which is programmed, exists at an organ, tissue, and cellular level and implicates *O*-acetylated sialic acids in cell development, homeostasis, and other functions [239]. This aspect of glycan structure and metabolism has relevance for the colonic mucins and in particular the Sd^a^ antigen.

A family of mucus binding proteins (MUBs) have been characterized in lactic acid bacteria*,* which are cell surface anchored effector molecules containing multiple mub domains. The precise pattern of glycan binding has not yet been resolved [225,240].

Carbohydrate binding modules (CBMs) are non-enzymatic domains found in many proteins that attach to glycan sequences in polysaccharides and glycoconjugates [241,242,243]. Over 69 families have been identified, indicating a wide range of glycan sequence recognition 

The design and use of array technology has offered a powerful method to examine the presence and function of glycan structures and this includes options to search for mucin related glycan epitopes, a few examples from a large literature are given as follows; [244,245,246,247,248,249,250,251,252,253]. Of particular interest are those arrays that correlate mucin glycan epitopes with bacterial binding [254,255,256,257,258,259]. Although a considerable range of glycans can be displayed and screened using this technique the conformation of the glycans on the surface of the chips remains a problem. Attachment of individual glycans can be achieved using a number of different methods and on different chip surfaces, see previous papers [244,245,247,249,252,253,255,260,261,262,263,264,265], however, this does not necessarily achieve the molecular conformation found *in vivo* when attached to proteins. Some improvements have been made using known glycans attached to peptides, where the normal in vivo conformation is more likely to be preserved [266,267,268]. A further problem is the density of attachment, which may not mirror the in vivo situation. Single glycan attachment, or clustered glycan attachment must conform with the biological arrangement in order to yield binding results that have genuine in vivo relevance [269]. Attempts have been made to address clustering, which is a feature of mucin *O*-glycans in the tandem repeat PTS domains of the mucins [250,270], but an array of *O*-glycans as found in mucins remains difficult to mimic. In spite of these problems, valuable information has been gleaned from glycan array screening.

As mucins represent a primary target for bacteria in the GI tract and other mucosal surfaces the production of a mucin microarray has been adopted for rapid throughput screening purposes [170,258,271,272]. Preparation of such mucin arrays relies on the prior purification of mucins from appropriate sources. As noted earlier, the preparation of mucins is demanding due to their high molecular weight and separation from other contaminant proteins, glycoproteins, and glycolipids. The available sources are also limited as many normal human mucosal tissues or their secretions cannot be obtained for ethical reasons and disease tissue will deliver abnormal mucin products. The use of cell culture is also dependent on the nature of the mucins produced by the cells. Most cell lines that produce and secrete mucins are cancer derived and as a result yield products that are also influenced by mutations and other cancer related changes including glycosylation. Finally, the attachment of mucins to the microarray plates will result in multiple attachment sites [258] and the conformation of the attached mucin is unlikely to mimic the in vivo situation, although no imaging studies have been reported. Atomic Force Microscopy (AFM) has provided images of purified mucins [273,274,275], but these also do not provide an ideal match for the in vivo mucins at mucosal surfaces. Force microscopy has been used for screening glycans structures. A range of different microscopic techniques have evolved and used to monitor glycans in various molecules including the mucins. This is an area where microscope design has driven the sensitivity and resolution of molecular imaging as well as yielding values for binding affinities [273,274,275,276,277,278,279,280].

A general appreciation of the biological significance of glycomics and the applications of glycoproteomics has grown in recent years [281]. This has led to increased awareness of glycan structure as a biological phenomenon requiring thorough assessment for all glycoconjugates, and glycoproteins and mucins in particular. It has opened the way for the involvement of synthetic chemical approaches to the strategic design of biological molecules with therapeutic application. This is not further detailed in this review.

Two recently developed technologies are worth mentioning at this point and although there is currently only limited application to glycobiology it is certain that they will attract attention in the immediate future. Firstly, the CRISPR-Cas9 genome editing methodology [282,283] has been used to the cell-specific delivery of the asialoglycoprotein receptor to hepatic cells [284]. The binding of the receptor to the cell surface, uptake through endocytosis, endosomal escape through endosome acidification and subsequent nuclear import has been achieved [284] and illustrates the power of this technology for application to mucosal surfaces. Secondly, the process of 3D bioprinting is being used in a variety of situations [285,286,287,288,289,290,291] and is an obvious target for mucosal surface bioengineering strategies. Significant interest in the pharmaceutical industry and development for high throughput screening bodes well for expansion of this technology in glycomics.

The wealth of glycomics information generated also prompted the development of methods to store and access the data. Glycoinformatics for processing and accessing the glycomics data has been reported [292,293,294].

## 8. Metabolism of Mucin Glycans

The metabolism of mucin glycans encompasses synthesis, degradation, and recycling. The synthesis of mucin *O*-glycans can be mapped to well-defined pathways in the ER and Golgi compartments of the cell, where the glycosyltransferases add the monosaccharides, one by one, to the growing *O*-glycan chain attached to the mucin peptide serine and threonine residues. The specificity of the glycosyltransferases governs the nature of the glycan chains synthesized and the complement of glycosyltransferases present in each cell determines the *O*-glycan core structures, backbone extensions and peripheral sialylation, fucosylation, and sulphation patterns. The absence of individual glycosyltransferases results in glycan structures, which may be shorter, less extended, or showing variations in sialylation, fucosylation, and sulphation. These events are dictated at the genetic level and form the basis for the type of *O*-glycans synthesized in any one cell [143,295,296].

The glycosyltransferases require an activated form of each monosaccharide to be transferred in addition to the growing *O*-glycan acceptor. Each monosaccharide exists as a nucleotide-sugar, and these donor molecules (See Table 6) are formed through standard pathways [297]. Active sulphate is also a substrate utilized in these pathways, while in the sialic acids, *O*-acetylation is mediated through acetyl-CoA transfer and *O*-methylation through *S*-adenosylmethionine and a methyltransferase.

The pathways leading to the nucleotide sugars derive from the hexose monophosphate pool, Glc-6P, and Fruc-6P. The hexosamine pathway is initiated by the amination of Fruc-6P with glutamine by glutamine:fructose amidotransferase (GFAT), which is feedback inhibited by UDP-GlcNAc, the end product of the pathway. UDP-GlcNAc is then further metabolized on the sialic acid pathway through two key enzymes, UDPGlcNAc 2-epimerase and ManNAc kinase, which act together in a bifunctional complex and lead to the formation of ManNAc-6P from UDP-GlcNAc [298,299,300]. This enzyme is feedback inhibited by the end product of this pathway, CMP-Neu5Ac [298]. UDP-GlcNAc may also be converted to UDP-GalNAc through the action of UDP-GlcNAc 4-epimerase and both of these nucleotide sugars are substrates for glycosyltransfer. The kinase generating GlcNAc-6P is subject to feedback inhibition by UDP-GlcNAc [301]. Free ManNAc enters the pathways after its conversion to GlcNAc by a specific GlcNAc 2-epimerase. 

**Figure 4:**
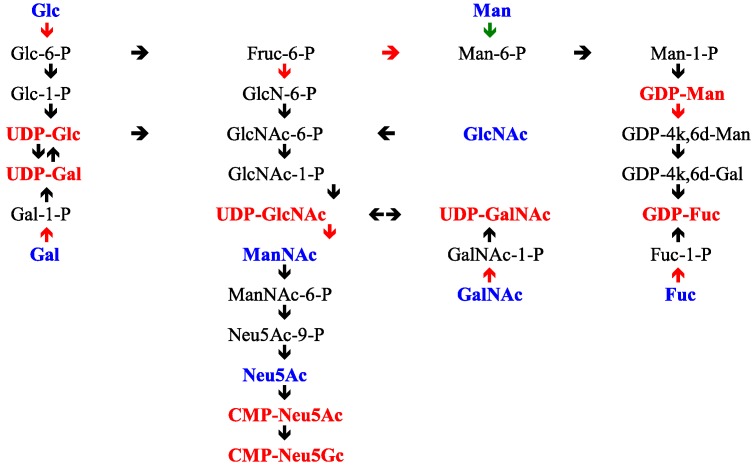
Feedback Inhibition on Glycan Activation Pathways. The Figure shows the known biosynthetic pathways relating to the formation and recycling/salvage of monosaccharides found in glycans. The nucleotide sugars are the end products of each pathway and are shown in red e.g., UDP-Glc. The individual reactions, which are subject to feedback inhibition, are shown with a red arrow, →. The individual monosaccharides found in glycan structures, and which are activated to the nucleotide sugars through the metabolic pathways, are shown in blue e.g., Glc. The black text and black arrows show the intermediate monosaccharides on the pathways and their conversion steps in the pathways. Abbreviations are as listed at the end of the paper.

Recycling or salvage pathways for monosaccharides ensure that optimal use is made of the monosaccharides generated during glycan degradation. The enzymes involved in these steps process monosaccharide intermediates and feed back into the main stream of metabolic pathways generating the nucleotide sugars. In this way d-GlcN, d-GalN, and d-ManN are re-*N*-acetylated to generate GlcNAc, GalNAc, and ManNAc. These N-acetylhexosamines are subsequently phosphorylated at the 1 position for GlcNAc and GalNAc, or at the 6 position for GlcNAc, GalNAc, and ManNAc. The phosphorylated sugars are part of the pathways leading to UDP-GlcNAc, UDP-GalNAc, and CMP-Neu5Ac. Free sialic acid is cleaved to ManNAc and pyruvate by the action of acylneuraminate pyruvate lyase [302,303,304], while ManNAc is recycled after epimerization to GlcNAc, or phosphorylation to ManNAc-6P. The control of these pathways is well integrated by end product feedback inhibition as noted above and shown in Figure 4.

UDP-GlcNAc is a crucial intermediate. It serves directly as a substrate for glycosyltransferases or it may be epimerized at the 2-position to generate *N*-acetyl-d-mannosamine, with loss of the UDP group, to enter the sialic acid pathway. It may also be 4-epimerised to yield UDP-GalNAc, another substrate for glycosyltransferases. The UDP-GlcNAc 2-epimerase is feedback controlled by CMP-Neu5Ac [299,300]. These patterns of monosaccharide metabolism serve to confirm the tight regulation that exists on the glycosylation pathways.

The initiation of *O*-glycan synthesis is through the action of a family of UDP-*N*-acetylg-alactosamine: polypeptide *N*-acetylgalactosaminyltransferases (ppGaNTases). These glycosyltransferases play an important regulatory role as their substrate specificity determines optimal glycosylation of the peptide ser/thr sites and influences the further extension of the *O*-glyca-n chains. Recognition of the mucin peptide ser/thr site and adjacent ser/thr sites that may already be glycosylated, coordinated action of the different isoenzymes to achieve optimal tot-al site glycosylation on any individual mucin peptide and participation of the lectin binding site found in the ppGaNTases emphasize the refinement of this initial transferase reaction [138,139,140,305,306]. Subsequent addition of sugars to form the range of *O*-glycans follows the established biosynthetic pathways [143,295,296]. Patterns for the biosynthesis of cores 1-4 are shown in Figure 1 and reveal the capacity for extension of these *O*-glycans to yield the wide range observed in the gastrointestinal tract mucins.

The *O*-glycan catabolic metabolic pathways allow the complete degradation of mucin *O*-glycans to individual monosaccharides. This is carried out though the action of glycohydrolases from the enteric micrbiota [307,308,309,310]. As the hydrolytic process is sequential and the *O*-glycans are covalently attached to the mucin peptide, the peripheral residues are the first targets for the glycohydrolases. The removal of sialic acids, fucose, and glycosulphate is necessary before the main *O*-glycan chains can be degraded. The complement of glycohydrolases required must cleave the different glycosidic linkages present for sialic acids (α2-3, α2-6 and α2-8/9), and fucose (α1-2, α1-3, α1-4 and α1-6). In addition, the sialic acids in the intestinal mucins are *O*-acetylated and the action of sialidases may be blocked by this modification, necessitating the action of an esterase prior to effective sialidase action. The same situation exists for the removal of fucose, attached through different glycosidic linkages to different sugars, therefore needing α-fucosidases with a range of specificities. In secretor individuals, where the blood group antigens are carried on the *O*-glycans these must also be removed before the remaining backbone and core structures can be degraded. Accordingly, α-*N*-acetylgalactosaminidase and α1-2 fucosidase are required to degrade A antigen and β-galactosidase and α1-2 fucosidase for B antigen. In H (O) individuals the α1-2 fucosidase is necessary.

Glycosulphate is often missed in analysis of glycohydrolases, but is a major chemical feature of gastrointestinal mucin *O*-glycans [98,162,163] and mucin specific glycosulphatases have been identified and measured [311,312,313,314,315,316,317,318,319]. Once the peripheral monosaccharides have been removed the remainder of the *O*-glycan chains can be hydrolyzed by the action of β-galactosidases and β-*N*-acetylhexosaminidases. The range of enzymes includes β-galactosidases specific for both β1-3 and β1-4 galactosides and N-acetylglucosaminidases cleaving β1-3 and β1-4 linkages in type 1 and 2 *N*-acetyllactosamine units. A β1-4 *N*-acetylgalactosaminidase will act on the Sd^a^ antigen, after the α2-3 Neu5Ac has been removed, and a specific α *N*-acetylgalactosaminidase removes the GalNAc attached to the mucin peptide ser/thr residues. The sequence of degradation is significant as most of the glycohydrolases described above act only on certain *O*-glycan structures. The Sd^a^ antigen is a good example, where the sialic acid must be removed before the β1-4 *N*-acetylgalactosamine can be released. This sequential strategy is analogous to the biosynthetic formation of the *O*-glycans and underlines the “reading” aspect of the sugar codes carried by the mucins. Thus, the absence of certain glycohydrolase activities will mean that incomplete degradation of the *O*-glycan may occur, leaving structures “available” for recognition by any of the glycan binding proteins that may be in the environment and possibly leading to events that will influence mucosal surface interactions and function. Recent work has highlighted the flexibility of mucin *O*-glycan degradation through a variety of metabolic pathways [197,320].

Catabolism of the nucleotide sugars is poorly studied. Hydrolysis of UDP-GlcNAc and UDP-GalNAc has not been reported in any detail and only one report gives information on specific enzymatic hydrolysis of UDP-GlcNAc to the nucleotide and free sugar [321]. Another possibility is that an alpha-*N*-acetylglucosamine phosphodiesterase, as described previously [322], could catalyze the same reaction, but there is no further work reported.

A CMP-Neu5Ac hydrolase has been reported located in animal liver plasma membranes and kidney [323,324,325] and unpublished work in our lab has confirmed the activity in human and rat colon. The enzyme may play a part in sialic acid metabolism, but there are no comparative studies to assess the general utilization of nucleotide sugars through their hydrolysis or glycosyltransfer.

In addition to the creation of *O*-glycans through the biosynthetic, recycling and salvage pathways the monosaccharides released may also be utilized as an energy substrate for the microbiota and this has been termed glycan foraging [66,142,309,310,326,327,328,329,330]. This process is beneficial to all bacterial strains that can internalize the monosaccharides [12,331,332,333] and is a further example of cross-feeding, where the combination of bacterial strains enables the degradation of mucin and other glycoconjugate glycan chains generating a pool of monosaccharides available to the total microbiota.

## 9. Glycan Expression When the Gastrointestinal Microbiota Is Removed

In order to better understand how the enteric microbiota in man can communicate with its host it would be valuable to remove the microbiota and assess the response of the host gastrointestinal mucosa. If mucin glycosylation is a “language” to enable dynamic “cross-talk” between the microbiota and the host, is it possible to test this hypothesis?

One option for studying the absence of the microflora in the gut is the use of animal models, in particular germ free mice. Such models have been widely used [334,335,336,337]. However, there are important caveats. Firstly, the microbiology of the mouse varies to that in the human gut [338], secondly, the glycobiology of the mouse gastric mucosa also varies to that in man [339], and finally there are immunological differences [340]. Together these caveats make comparison with the human gut difficult and unpredictable. Accordingly, research in this area requires careful consideration of the specific research focus before use of the model can be adopted.

Gastrointestinal surgery offers an option to carry out such an experiment. In cases where patients have gastrointestinal disease the surgeons opt to isolate the colon from the normal faecal flow. This has been termed faecal diversion [341,342,343]. Only one of the hospitals where this procedure is used has examined the mucosal glycobiology. The Department of Surgery at the Bristol Royal Infirmary has designed such an experiment. Tissue and biopsy samples were taken from the colon at the initial operation when the faecal flow was isolated and subsequently at the second operation to reestablish the normal faecal flow. Samples were taken from normal mucosa, close to the anastomosis site at each operation. In total 58 patients with no diversion and 49 with diversion were studied, of these 30 non-diverted and 17 diverted had ulcerative colitis (UC); 9 non-diverted and 17 diverted had Crohn’s Disease (CD) and 19 non-diverted and 15 diverted had non-IBD disease. All of the patients in the study gave written permission for the samples to be taken and used for research. The study was conducted in accordance with the Declaration of Helsinki, and ethical approval for all experiments was gained from the United Bristol Hospital Trust Ethics Committee.

Pathological analysis of the tissue sections was carried out by Prof. Bryan Warren. Tissue observation and scoring was carried out with three observers. Tissue sections were prepared and tested histologically with standard histochemical stains, Diastase periodic acid Schiff (PAS), and Alcian Blue (AB), the PAS/AB stain to identify the pattern of acidic and neutral mucins; the High Iron diamine (HID)/AB stain for sulphated and carboxylated, sialylated mucins; and the mild PAS (mPAS) stain to identify sialylated and not sulphated mucins, this was performed with and without a prior saponification step to remove the *O*-acetyl esters normally present on the colonic mucins and which block the periodic acid oxidation of the sialic acids. These methods have been described previously [344].

In addition, to screen for the presence of mucins and mucin glycans of various types a series of lectins and antibodies were used to test the tissue sections. These are shown in Table 7.

Those reagents binding to sialic acids were tested with prior mild saponification to allow for the possible interference of *O*-acetylation. Note that this is the case for the Sd^a^ antigen, which is *O*-acetylated in the human colon, see Figure 4.

Metabolic labeling with ^3^H-d-glucosamine and ^35^S-sulphate was carried out for all samples, on the same day as they were collected, to probe for active mucin biosynthesis. This was carried out according to a procedure established in our laboratory in Bristol [368]. Statistical analysis for all of the data reported in this review was performed using Unistat software. Non-parametric data were compared using the Mann–Whitney *U*-test and matched pairs were compared using the Wilcoxon Signed Rank test.

The results of this histological analysis revealed a selective pattern of changed mucin glycosylation in response to the removal of contact with the faecal micobiota. Testing for the mucin genes themselves, MUC1, MUC2, MUC3A/B, MUC4, MUC5AC, MUC5B, MUC11, and MUC13 show no differences between the diverted and non-diverted patients, see Table 5, indicating that the glycans carried by the mucins may be the target for the transformations observed. As well as the mucin gene probes the standard histochemical stains PAS/AB, HID/AB and mPAS showed no differences and neither did the metabolic labeling with ^3^H-d-glucosamine and ^35^S-sulphate [369,370]. These methods detect a range of different epitopes and do not appear to have the sensitivity to indicate more subtle changes. More specific reagents need to be used for screening and here the choice is much larger. Ideally an array approach is required and this is the current mode of study, with array technology using lectins [371,372,373] and antibodies [212,374,375,376]. This approach is in addition to the glycoarray platforms discussed earlier.

**Figure 5:**
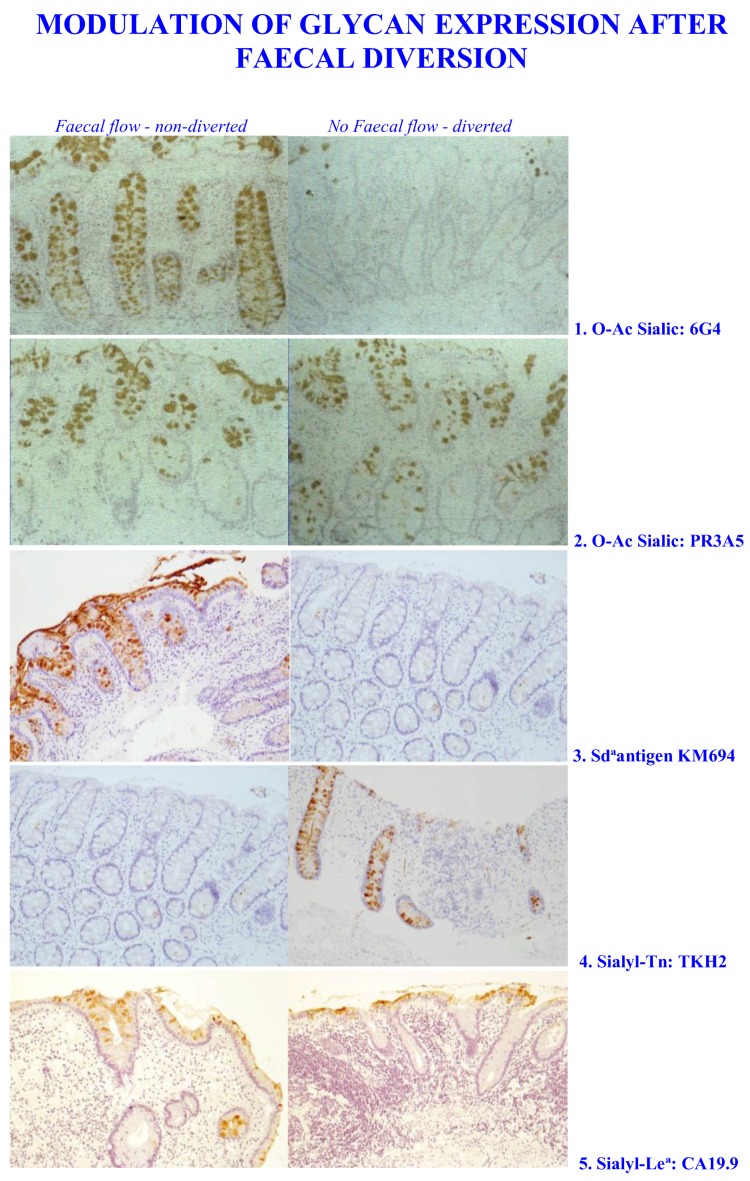
Modulation of Glycan expression after Faecal Diversion. Human colic tissue samples were stained using the antibodies for the epitopes indicated. Note that 1 and 2 represent different antibodies that bind to *O*-acetylated sialic acids.

Screening for mucin glycans revealed a selective mucosal response to the removal of the microbiota. Increases in the expression of sialyl-Tn and Sialyl-Le^a^ were observed, while loss of sialic acid *O*-acetylation with the 6G4 antibody and the Sd^a^ antigen with the KM 694 antibody and the DBA lectin, were found, see Table 7 and Figure 5. Testing for a range of other glycan structures showed no change in the diverted and non-diverted groups.

Analysis of sialic acid *O*-acetylation revealed an interesting pattern depending which reagent was used. The mPAS method showed no differences, neither did the PR3A5 antibody, however a loss was detected with the 6G4 antibody. The specificity of the PR3A5 and 6G4 antibodies has not been determined, but the result indicates that there is a specific population of *O*-acetylated sialic acids in colonic mucins that is deleted as a result of faecal diversion (Table 7 and Figure 5). This may relate to the Sd^a^ antigen, which would represent a selective population of glycan sialic acids, which are *O*-acetylated and are known to be deleted in colorectal cancer (see below). This remains to be examined. The situation for sialic acid non-*O*-acetylators remains to be addressed.

## 10. Aberrant Mucin Glycosylation and Disease in the Gastrointestinal Tract

There is a considerable literature on the glycobiology of gastrointestinal disease and only a few examples are given here to illustrate how defects in reading the sugar code by bacteria may impact the host. Recent developments have seen the application of array technology, including glycoarrays, to the screening of disease patterns and the identification of abnormal glycan sequences associated with these changes [254,268,377,378,379].

As the mucins contribute a major part of the mucosal barrier, both as secreted adherent mucus gels and glycocalyx membrane-associated mucins, they may be involved at several levels. Firstly, the loss of the secreted mucus gel removes the normal barrier, keeping bacteria and luminal contents away from the apical mucosal cell surface. In addition, there appears to be a feedback regulation for the presence of a mucus gel at the colonic mucosal surface, where absence of the mucus gel triggers events that lead to intestinal mucosal malfunction. This has been demonstrated in mice where the Muc2 gene has been deleted [380,381,382]. Secondly, the membrane-associated mucins become accessible to the luminal microbiota and will be subject to molecular manipulation through (1) modification by secreted and membrane linked bacterial enzymes, including proteases, peptidases, and glycohydrolases; (2) stimulation of abnormal mucin gene expression and glycosylation pathways; and (3) inappropriate triggering of other signaling pathways. Therefore, the mucins may contribute to disease pathology through deletion or expression of abnormal MUC genes, changes in the proportion of mucin genes expressed or the production of mucins with abnormal glycosylation.

### 10.1. Bacterial Strains and Gastrointestinal Disease

*Helicobacter pylori* is a common stomach infection in humans and stomach is associated with gastritis and duodenal ulcers and is linked to the development of gastric cancer [383]. It is found in the mucus gel layer and contact with the host stomach epithelium leads to disease [272,384]. It possesses a number of adhesins, which bind to glycan sequences in the secreted mucus itself and also with the surface membranes of the stomach mucosal cells. The adhesins include BabA which binds to Lewis^b^ (Fucα1-2Galβ1-3(Fucα1-4)GlcNAcβ-) [385], SabA, which binds to sialyl-Lewis^x^ (Neu5Acα2-3Galβ1-4(Fucα1-3)GlcNAcβ-), [386] and LabA which binds to *N*,*N*′-diacetyllactosediamine GalNAcβ1-4GlcNAc [387]. All of these glycans are carried by MUC5AC in the stomach. *H. pylori* is very adaptable to the variable stomach environment and shows rapid genomic change through mutation and homologous recombination adjusting adhesin production and host glycan expression.

*Campylobacter jejuni* is an intestinal Gram-negative, flagellate pathogen, responsible for global nutrition-based bacterial gastroenteritis [388,389,390]. It is a commensal in chickens and they are a significant reservoir for human infections [391,392]. It has also been linked with the development of Guillain–Barré syndrome, a progressive neuromuscular paralysis [390,393]. Evidence for N-linked glycosylation genes has been reported [394,395,396] and *N*-glycans with a heptasaccharide structure, GalNAcα1-4GalNAcα1-4(Glcβ1-3)GalNAcα1-4GalNAcα1-4GalNAcα1-3Bac, where Bac is bacillosamine, 2,4-diacetamido-2,4,6-trideoxyglucose, have been shown [397,398].

*Campylobacter jejuni* lipopolysaccharide is sialylated and shows molecular mimicry with host gangliosides. The binding of sialoadhesin [399] and siglec-7 [400], indicates binding to sialylated glycans. This is further supported by the detection of sialic acid synthase genes in the *C. jejuni* genome [401], identification of a bifunctional sialytransferase forming α2-3 links and α2-8 links, as found in gangliosides [402] and detection of a sialic acid *O*-acetyltransferase [403,404]. The flagella protein flagellin is *O*-glycosylated and the glycans contain a variety of pseudaminic acids, Neu5Ac analogues with an extra *N*-acetyl group at position 7 [405,406]. Further, fucosylated glycans in milk have been shown to protect against *C. jejuni* infections [407] demonstrating roles for fucose in the overall pattern of glycan interactions. 

Attachment of *C. jejuni* to the human intestinal mucosa includes binding to MUC2, which also stimulates virulence gene expression [408]. This abundance of glycan related interactions underlines the flexibility of glycosylation as an information medium utilized by the bacterium. Indeed, the ready availability of *C. jejuni* strains has been adopted by the synthetic chemists to isolate many glycoenzymes for preparation of glycans in general [409,410,411,412,413].

A wide variety of *Escherichia coli* strains have pathogenic activity in the human intestinal tract. These have been termed enteroaggregative, diffusely adherent, enteropathogenic, enterohaemorrhagic, enteroinvasive, and enterotoxigenic. These strains use several types of pili to adhere to host mucosal surfaces and a number of fimbral adhesins, which bind the mucosal glycans have been detected. The tip of type 1 pili have FimH, which binds to mannose on host cell surfaces [414,415]. These are tissue specific and while the large intestinal FimH binds to monomannosylated glycans, the FimH in the urinary tract bind to oligomannose host glycans [416]. Glycan analogues have been used therapeutically to block *E. coli* binding and infection [417,418]. Other *E. coli* adhesins include PapG, which binds to the disaccharide Galα1-4Gal [419] and UclD, which binds to *O*-glycans on large intestinal mucosal cells. Treatment of the cells with an *O*-glycosidase abolished the binding, but the glycan receptor structures have not yet been identified [420].

*Clostridium difficile* occurs as a recurrent infection in both IBD and non-IBD patients and is a leading cause of antibiotic-associated diarrhea [421]. The glycan epitopes of the *C. difficile* cell wall polysaccharide have been identified through glycoarray screening, and potential short glycan analogues identified as possible vaccine targets [422].

*Ruminococcus gnavus* is a commensal anaerobe and also belongs to the mucin degrader category of enteric bacteria in man. It is found in over 90% of the population. However, the ability to degrade mucins is strain specific [423]. *R. gnavus* ATCC 35,913 has a Nan cluster, including an intramolecular trans-sialidase, RgNanH, which contains a GH33 catalytic domain and a sialic acid-binding CBM40. It releases sialic acids from substrates as 2,7-anyhydro-Neu5Ac and is able to grow with this product as a sole carbon source [308]. In addition, it codes for α-galactosidase, which can degrade the B-antigen [424,425]. IBD is associated with a 4-fold increase in *R. gnavus* and a 100 fold increase in *Ruminococcus torques* in both UC and CD [426,427]. There is also evidence that this occurs in infant disease [428]. R. gnavus E1 influences the expression of colonic glycoconjugates, probably including mucins, in mucosal Goblet cells. R. gnavus E1 induces individual mRNAs for mucins including MUC2 and also for glycosyltransferases in both mice and HT-29-MTX cells through the action of a soluble, heat stable, low molecular-weight (<3 kDa) peptide [429]. It is clear that *Ruminococcus* spp. in the gut have a major role in mucin metabolism.

### 10.2. Necrotising Enterocolitis (NEC) 

Necrotizing enterocolitis is a condition known for premature, low-birth-weight infants. Its aetiology is multifactorial and details of the pathogenesis are still unclear [430]. The compromised immature mucosa in these neonates leads to the binding of bacteria and the initiation of mucosal infection and mucus barrier damage. The neonates are born with no exposure to the microbiota and the initial contact comes from the mother and the external environment [431].

The stability of the mucus barrier and the innate defense mechanisms rely on the secretion of intact, gel forming mucus and the availability of mucosal immune proteins, including immunoglobulins and the trefoil factor peptides. Together these factors normally ensure mucosal cell integrity and epithelial homeostasis through restitution and regeneration processes. Part of the risk is linked to the immature state of the mucosa at this very early stage of life. A role for the mucins in the pathogenesis of NEC is indicated by the depletion of Goblet cells seen in the mucosa of neonates with NEC [432,433,434] and this is expected to represent a reduction of MUC2 in the secreted mucus gel. Evidence for abnormal microbiota causing NEC has been proposed [435] and probiotics (*Bifidobacillus* and *Lactobacillus* strains) have been used in the treatment of the disease [436,437,438]. 

A number of glycan-based prebiotics have been shown to alleviate the disease in man and animals [439,440]. Oligofructose [441] and disialyllacto-*N*-tetraose [439] have been used in the quail, sialylated galacto-oligosaccharides, disialyllacto-*N*-tetraose, and 2′-fucosyllactose are effective in rodent disease [440,442]. In addition, the exposure of the T-antigen (Galβ1-3GalNAc) on red cells by the action of bacterial sialidase has also been reported in NEC [443]. Examination of NEC glycobiology is still outstanding, but it is clear that recognition of mucin glycoarrays is an essential part of the mucosal defensive barrier and the action of prebiotics also implicates recognition of glycan sequences. Understanding the roles of the mucins and their glycosylation will allow disease mechanism and therapy to be improved [444].

### 10.3. Inflammatory Bowel Disease (IBD)

The inflammatory bowel diseases, ulcerative colitis (UC) and Crohn’s Disease (CD) cause mucosal damage in the colon. They are characterized by modification or loss of the mucus layer on the surface of the mucosal cells. The resulting contact between the luminal bacteria and the mucosal cells triggers an inflammatory response characteristic of the diseases. The aetiology remains unknown, although proposals have been made that those individuals that are genetically susceptible generate an abnormal immune reaction to the resident microbiota [445,446]. Much effort has been made to identify the genetic nature of these individuals and over 150 IBD genetic susceptibility loci have been identified of which 70% are shared by UC and CD patients [447]. IBDs are multifactorial diseases because of the range of factors, which normally contribute to the formation and maintenance of the mucosal defensive barrier. Defects in one or more of these factors can therefore lead to a general effect where bacterial contact with the mucosal cells occurs and inflammation is triggered. The loss of the mucus layer in murine colitis has been shown to enable bacteria to penetrate the remaining aberrant, gel barrier and contact the mucosal cells, leading to the characteristic inflammatory responses [448].

A role for mucins and glycosylation in IBD has been well established, but remains a complex issue in line with the multifactorial nature of the diseases. In addition, the diseases show active and inactive phases and there is evidence that the patterns of mucin expression, including glycosylation, revert to normal in the inactive phases and this is also observed in disease remission [449]. Accordingly, most studies have been directed at patients with active disease.

The normal intestinal pattern of MUC genes is altered in both UC and CD [29,96,450]. Ileal expression of MUC1, MUC3A/B, and MUC4 showed normal patterns in UC and CD patients [344,451,452,453]. Transcriptional analysis showed reduced mRNA for MUC1, MUC3A/B, and MUC4 [344] and increased levels for MUC13 [26]. A reduction in MUC17 protein was also found [454]. The secreted gel-forming MUC2 showed normal hybridization patterns for mRNA in UC and CD patients with active disease, but was reduced if the secreted mucin product was assayed [455].

Further studies showed decreased mRNA in UC and no change in CD [451,453]. Evidence for neo-expression of MUC5AC was found in adults [456] and children with IBD [457]. A subpopulation of cells in the colon have been shown to express MUC5B [458], but analysis of MUC gene expression in UC showed no expression of MUC5AC, MUC5B, or MUC6. A reduction of MUC2 was seen in tissue adjacent to ulceration and MUC1 was upregulated in severe disease [344].

Ethnicity is also a factor impinging on IBD. South Asians compared to European individuals have a lower risk of cancer due to UC [459,460] and African Americans do not show any difference to disease activity compared to Caucasians [461]. Whether this is due to a genetic trait remains to be demonstrated.

The enteric microbiota in IBD has been a focus of attention, both in the detection of disease related alterations and in the potential therapy of faecal transplantation. Regulation of the microbiota is the pivotal feature in the maintenance of a normally functioning GI tract in mammals [462,463,464]. IBD patients show a decrease in gut commensals, including *Akkermansia*, *Clostridium* IXa and IV groups, *Bacteroides*, and *Bifidobacteria* and an increase in sulphate-reducing bacteria and *Escherichia coli*. The resulting dysbiosis leads to defective innate immunity and killing of certain bacteria and loss of commensals, which may be involved in microbiota-mediated protection [426].

A number of reports have shown altered glycosylation of mucins during IBD [26,449,465,466]. As IBD is also seen as a possible intermediate stage in the development of intestinal cancers many of these changes are observed in both IBD and colorectal cancer. As already detailed before, the glycosylation of the intestinal mucins is characterized by relatively large glycan chains containing more than 5 monosaccharides and terminated with sialic acids, fucose, and sulphate moieties. The loss of the mucin core 1 and core 3 structure in mice and man results in reduced MUC 2 synthesis. This may be due to the loss of the core 1 and core3 synthases [467,468,469,470,471] or depletion of the β1-3-*N*-acetylglucosaminyl-transferase. Loss of the β1-6-*N*-a-cetylglucosaminyl-transferase blocks the formation of the core 2 structure and also leads to I-BD in mice.

Increased α2-6 sialyltransferase activity in IBD leads to abnormal synthesis of sialyl-Tn (Neu5Acα2-6GalNAc-) and prevents the normal extension of the colonic mucin *O*-glycan chains. Sialyl-Tn is a well know marker of both IBD and colorectal cancer [449,472]. Perhaps as a result of this change the glycans on MUC2 in UC are generally shorter and significant levels of Tn (GalNAc-ser/thr) and TF antigen (Galβ1-3GalNAc-ser/thr) are seen [449,466]. Alterations in glycosyltransferase may also arise due to alterations of the Golgi lumen pH in mucosal Goblet cells [473].

No changes in ABO or Lewis antigens were reported in UC [419], but decreases in the amounts of more complex glycans such as Neu5Acα2-3Galβ1-3(Fucα1-4)GlcNAcβ1-3(Neu5Aca2-6)GalNAc-, Neu5Acα2-3(GalNAcβ1-4)Galβ1-4GlcNAcβ1-3GalNAc- (an Sd^a^ antigen related glycan) were seen. In addition sulphated structures, e.g., SO_3_-Galβ1-3(Fucα1-4)GlcNAcβ1-3(Neu5Acα2-6)GalNAc-, were also depleted in agreement with metabolic labeling studies in UC patients with active disease [370,474].

The *O*-acetylation of the sialic acids is deleted in both UC and CD. This has been detected using histochemical methods [189,190] and also with antibody and chemical methods [191,358,368]. The data suggest that the loss of *O*-acetylated sialic acids is selective and site specific and is recovered on remission of disease. The antibodies PR3A5 and 6G4, which both detect *O*-acetylated sialic acids, show different staining patterns throughout the human colon [358,359]. The histochemical methods do not discriminate between different glycoprotein or glycolipid carriers and the PR3A5 antibody appears to react with total *O*-acetylated sialic acids, however the 6G4 antibody stains for a selective population. In faecal diversion it is only the 6G4 antibody which shows a loss of expression, with both mPAS and PR3A5 staining remaining constant, see Table 8. Note that these changes are similar to those mentioned earlier for faecal diversion.

Further evidence for changes in sialic acid metabolism were found in faecal extracts from IBD patients. Increased acylneuraminate pyruvate-lyase activity was found, but sialidase activity did not differ from normal. This suggests that free sialic acids may be a target for scavenging by the bacterial flora in IBD [475]. Thus the metabolism of sialic acids is strongly implicated as a target for change in IBD.

Treatment of IBD relating to the microbiota has been a major issue. Probiotics may alter the enteric microbiota and have been selected on the basis of experience with human and animal models. *Lactobacillus* spp., *Bifidobacteria* strains, *Akkermansia muciniphila*, and other non-pathogenic commensals have been used [476,477,478]. Probiotics show some benefits in IBD, but require regular administration. However, the variable results obtained emphasize the wide range of individual characteristics, which contribute to normal health.

Faecal microbiota transplantation has also been adopted in human and mouse models [479] and has been shown to be effective in dealing with *Clostridium difficile* infect in IBD [480,481,482].

Faecal diversion has also been employed in the treatment of UC and CD and has an impact on mucosal glycosylation as already noted above in the section “Glycan Expression when the Gastrointestinal Micobiota is removed”. Sialic acid *O*-acetylation is deleted [483], together with the Sd^a^ antigen and increases in the expression of sialyl-Tn and Sialyl-Le^a^.

Additional features associated with IBD include ER stress [484]. ER stress triggers the unfolded protein response and is implicated as a disease mechanism leading to IBD [484]. The failure to resolve ER stress though Paneth and Goblet cells results in inflammation. Autophagy is also a factor in the development of IBD, especially CD, and has been linked with ER stress and the NOD genes [485].

### 10.4. Colorectal Cancer 

The development of colorectal cancer may follow the events occurring during IBD and many of the glycosylation changes found in IBD persist in colorectal cancer. Cancer is also characterized by a progression through adenomas to carcinomas. A defective and reduced mucus barrier allows the microbiota to make contact with the mucosal epithelial cells and lead to an inflammatory response. The governing Th1/Th17 inflammatory response results in an overexpression of a modified and abnormal glycocalyx, disruption of the normal cell–cell contact at tight junctions, and further increases in permeability and inflammation. The changes fit well with the proposed adenoma-carcinoma sequence [486,487].

Several mucin based events have been noted which are characteristic of colorectal cancer. Reduction or loss of MUC2 diminishes the major gel forming mucin at the mucosal surface and this has been clearly shown in the mouse model, where the Muc2^−/−^ mouse shows spontaneous tumor formation [382]. Deletion of MUC3 is also a feature of adenomas [488]. The initiation of colorectal cancer is associated with neo-expression of the secreted mucin MUC5AC, low levels of MUC6, and overexpression of MUC20 [489,490,491,492]. MUC1 is increased in colorectal cancer [493].

Normal colorectal mucin glycosylation is characterized by high core 3 and core 4 based glycans, glycans with 8-9 monosaccharides, branched glycans, high sialylation, sialate *O*-acetylation, and high sulphation [162,163,494]. Abnormal glycosylation patterns are associated with different stages from adenoma to carcinoma in the colon. These include reduced expression of core 1, core 3, and core 4 linked glycans [20,495,496], increased sialylation [143,497] and decreased sulphation [498,499,500,501]. The synthesis of the core structures is a vital step in *O*-glycan biosynthesis and loss of core 3 and core 4 synthases has been reported in colorectal cancer [191,468,495]. This fits well with the patterns of glycans detected in mucins from colorectal tumors [494].

Increased levels of sialyl-Tn in colorectal cancer have been well documented [472,502]. They can arise due to a number of possible mechanisms. Firstly, if the glycosyltransferases that form and elongate the core1 and core 3 structures are absent, only the Tn and sialyl-Tn structures can be formed. Secondly, upregulation of the sialyltransferase *ST6GALNAC1* leads to the formation of sialyl-Tn, which is a “dead end” glycan as there are no glycosyltransferases known which can act on this disaccharide. The increased *ST6GALNAC1* competes against the core 1 and core 3 synthases to prevent the normal levels of mucin cores being formed [20,496,503,504]. The faults may arise due to mutations in the glycosyltransferases to generate inactive enzymes, alternatively subcellular targeting of the transferases to the wrong ER and Golgi compartments will prevent their action on the normal substrates to complete the expected extended mucin glycans. In addition, modification of the pH of the Golgi lumen may prevent optimal action of the glycosyltransferases [473]. As noted for IBD, this results in a depletion of the extended glycans normally found in colonic mucins and the appearance of incomplete, truncated glycans such as the Tn-antigen (GalNAcα-ser/thr) [502,505], and TF-antigen (core 1, Galβ1-3GalNAc-) are hallmarks of these cancer related changes [506,507]. Other changes in cancer mucin sialylation include an increase in core 3 sialyl-Le^x^ NeuAcα2-3Galβ1-4(Fucα1-3)GlcNAcβ1-3(NeuAcα2-6)GalNAc- [508,509] and loss of the loss of the Sd^a^ antigen Gal*N*Acβ1,4(Neu5Acα2,3) Galβ1,3/4Glc*N*Acβ1,3Gal*N*Ac- [188]. These changes are modulated by the specific sialyltransferases [510]. The formation of the Sd^a^ antigen under normal conditions competes with the biosynthesis of the sialyl-Lewis antigens, which only occur at low levels. Dramatic loss or down regulation of B4GALNT2 has been shown in colorectal cancer [23], leading to increased formation of the sialyl-Lewis antigens, which have been employed as biomarkers for colorectal cancer [188,511].

The *O*-acetylation of sialic acids is lost early in colorectal cancer [191,512,513,514]. This has been correlated with the loss of the *O*-acetyltransferase specific for the sialic acids (OAT) [191]. These *O*-acetyl esters protect the sialic acids that carry them against sialidase activity. This may be relevant in the colonic lumen, where the enteric microbiota expresses a number of sialidases [63,191,515,516]. The recent improved assessment of sialic acid *O*-acetylation using the nidovirus virolectins implicates *O*-acetylated sialic acids in cell development, homeostasis, and other functions [239]. As it has previously been impossible to screen for a differential pattern of *O*-acetylated sialoglycoproteins in the colon, the role of various sialoglycoproteins could not be assessed. The use of the nidovirus virolectins opens the way to test for the possible functional roles of *O*-acetylated sialoglycoproteins and the potential impact of their loss in colorectal cancer.

Glycosulphation is reduced or lost in colorectal cancer and this is evident for the HID histochemical staining method [499], metabolic labeling with ^35^S-sulphate [369,370,473], and chemical techniques showing a decrease in sulpho Le(x) SO_3_-3Galβ1-4(Fucα1-3)GlcNAcβ1-3(NeuAcα2-6)GalNAc- [494,508]. The biological impact of sulphation and the significance in disease is poorly understood and although the depletion of colonic mucin sulphation in colorectal cancer is well known there is no information regarding the relevance of sulphation on different mono-saccharides and on different positions on these sugar residues. The patterns of glycosulphation remain to be linked to specific functions of the sulphoglycoproteins and also with the sulphotransferases which form the mature products [498] and the glycosulphatases which are present in the enteric microbiota and which may play a role in colorectal cancer and colorectal disease in general [63,312,315,319,515,517]. Evidence for glycosulphatases with specificity for different glycosulphated glycans has been presented [313].

## 11. Mucin Glycans; Future Prospects

It will be clear from this review and elsewhere that many facets of mucin glycosylation remain to be examined in detail [518]. This final section provides a brief, bullet point assessment of some of these targets for future research.

In general terms technological advances will drive the progress. Mass spectrometry (MS) has been a fundamental method for the mapping of glycosylation patterns and has been developed in a variety of modes [213,518]. Combination of MS with other techniques such as high-pressure liquid chromatography (HPLC) and nuclear magnetic resonance (NMR), have broadened the application of the study of glycans and glycoconjugates. The design of new bio-orthogonal labeling methods continues to improve the assessment of glycan expression in real time [519,520]. Other fields where rapid improvements are apparent are X-ray crystallography, electron imaging methods such as cryo-electron microscopy and force microscopic techniques—atomic force microscopy (AFM) [273,274,275,276]. Current emphasis is on the simplification of techniques to allow high throughput approaches for broader analysis.

A number of fundamental questions remain to be addressed, see Hart and Varki [518]. Issues that are related to the topics discussed in this review are briefly listed below:

1. The attachment of *O*-glycans to either serine or threonine residues in mucin tandem repeat domains has been identified as a regulated biological event. The significance of this biochemical configuration deserves wider exploration. Methods to carry out such screening are available.
2. Why does fecal diversion trigger changes that are also seen in cancer?
3. Nucleotide sugar metabolism is fundamental to normal glycosylation processes. However, the links with catabolic pathways and their integration with biosynthesis are still poorly understood and should be examined more closely at cell, tissue and organ levels.
4. Different populations of *O*-acetylated sialic acids in colonic mucins are evident in the colorectal mucosa as detected by the PR3A5 and 6G4 antibodies. These patterns should be reexamined using the nidovirus probes reported recently in order to obtain more precise information regarding their biological functions. In addition, the identification and biological significance of non *O*-acetylators should be examined in order to understand the importance of sialic acid *O*-acetylation.
5. The Sd^a^ antigen is implicated as a major sialoglycan present in colonic mucins, which is normally *O*-acetylated, but is lost in colorectal cancer and in fecal diversion. It is clear that bacteria talk to this mucin glycan. Does the Sd^a^ antigen represent a discrete group of *O*-acetylated sialic acids?
6. Mucin sulphation is poorly studied. A combination of chemical and immunohistological studies is needed to address the expression patterns in normal health and the implications of specific deletions in disease.
7. In IBD changes relating to the age of patients, their ethnicity, disease severity deserve further investigation. In addition, the composition of the microbiota during development should be examined for IBD patients.

It is plain that the wide scope of issues linking mucins and their glycosylation with the microbiota continues to excite researchers in this incessantly developing field.

## Figures and Tables

**Table 1:** The Mucin (MUC) Gene Family.

MUC Gene	Chromosome	Tandem Repeat Size	N-Terminal Signal Sequence	Gastrointestinal Tract Location
*Membrane Associated Mucins*				
MUC1	1q21	20	√	Stomach, duodenum, ileum, colon
MUC3A/B	7q22	17	√	Small intestine, colon
MUC4	3q29	16	√	Small intestine, colon
MUC12	7q22	28	√	Colon
MUC13	3q21.2	27	√	Small intestine, colon
MUC15	11p14.3	none	√	Small intestine, colon
MUC16	19p13.2	156	√	Not expressed
MUC17	7q22	59	√	Stomach, duodenum, colon
MUC20	3q29	18	√	Colon
MUC21	6p21	15	√	Colon
*Secreted gel-forming mucins*				
MUC2	11p15.5	23	√	Jejunum, ileum, colon
MUC5AC	11p15.5	8	√	Stomach
MUC5B	11p15.5	29	√	Salivary glands
MUC6	11p15.5	169	√	Stomach, ileum
MUC19	12q12	19	√	No reports for GI tract
*Secreted non gel-forming mucins*				
MUC7	4q13-q21	23	√	Salivary glands
MUC8	12q243	13/41	√	Not expressed
MUC9	1p13	15	√	Not expressed

The chromosome location, size of the tandem repeat domain, confirmation of an N-terminal sequence, and expression pattern in the gastrointestinal tract are shown.

**Table 2:** Mucin peptide domains.

Peptide Domain Type	Mucin	Mucin Type	Peptide Domain Function
Cysteine rich CYS domains	MUC2, MUC5AC, MUC5B, MUC19	Secreted	Non-glycosylated multiple copy domains adjacent or interrupting tandem repeat domains. Important for various mucin–mucin interactions.
Cysteine Knot	MUC2, MUC5AC, MUC5B, MUC6, MUC19	Secreted	Involved in dimerization.
Von Willebrand Factor D (D1, D2, D’, D3)	MUC2, MUC5AC, MUC5B, MUC6, MUC19	Secreted	Mediate oligomerisation located at N- & C-terminus D3 is directly active in polymerization.
Von Willebrand Factor D (D4)	MUC2, MUC5AC, MUC5B, MUC6 MUC4	Secreted & Membrane-associated	Located N-terminally to the D4 is located C-terminally to the VNTR domains, contains the GDPH autocatalytic cleavage site.
Cytoplasmic Tail	MUC1, MUC3A/B, MUC12, MUC13, MUC16, MUC17, MUC21	Membrane-associated	Located on the cytoplasmic side of the cell surface membrane. Contains phosphorylation sites involved in signaling. MUC3, MUC12, and MUC17 have PDZ binding motifs
SEA (Sperm protein, Enterokinase & Agrin)	MUC3A/B, MUC4, MUC12, MUC13, MUC17, MUC21	Membrane-associated	Protein binding properties. Contains autocatalytic proteolytic cleavage site.
EGF (Epidermal Growth Factor)	MUC1, MUC3A/B, MUC12, MUC13, MUC17	Membrane-associated	Mediate interactions between mucin subunits and ERBB receptors.
Transmembrane	MUC1, MUC3A/B, MUC4, MUC12, MUC13, MUC16, MUC17, MUC20, MUC21	Membrane-associated	Membrane-spanning sequence typical for membrane proteins
GDPH autocatalytic proteolytic site	MUC2, MUC4, MUC5AC	Secreted & Membrane-associated	Autocatalytic site cleaving between GD and PH residues
Proteolytic cleavage site	MUC1, MUC3A/B, MUC4, MUC12, MUC13, MUC16, MUC17	Membrane-associated	Found in MUCs with the SEA domain and in MUC16

The major mucin peptide domains are indicated for each of the secreted and membrane-associated mucin genes. An indication of their function is summarized. In addition to the conventional mucin forms, there are similar molecules that have been given names such as mucin-like, see previous papers [54,55,56]. These molecules are different to the mucin family shown in Table 1 and are not considered further in this review.

**Table 3:** Mucin Core and Backbone Repeat Glycan Structures.

Core Type	Structure
1	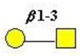 Galβ1-3GalNAc
2	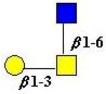 Galβ1-3(GlcNAcβ1-6)GalNAc
3	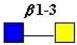 GlcNAcβ1-3GalNAc
4	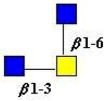 GlcNAcβ1-3(GlcNAcβ1-6)GalNAc
Backbone Repeat	Structure
Type 1	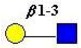 Galβ1-3GlcNAc
Type 2	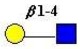 Galβ1-4GlcNAc
Poly *N*-acetyllactosamine type 2	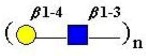 (Galβ1-4GlcNAcβ1-3-)_n_
Branched *N*-acetyllactosamine type 2	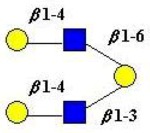 Galβ1-4GlcNAcβ1-6 Galβ1 Galβ1-4GlcNAcβ1-3

The range of basic mucin glycan core and backbone structures are shown. The details of the abbreviations and symbols are indicated at the end of the paper.

**Table 4:** Protein Glycosylation Patterns.

Protein Carrier	Glycan Structure
Glycoproteins *N*-Glycans	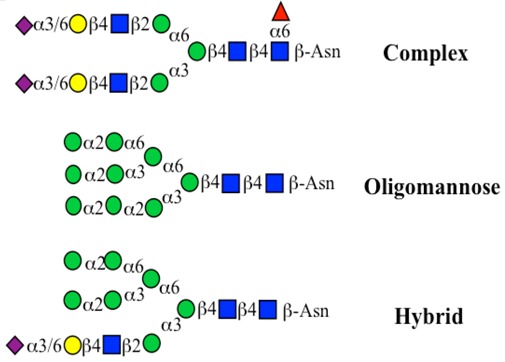 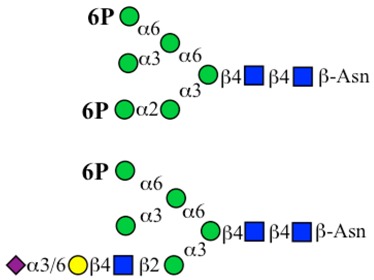 Mannose 6-phosphate glycans
Glycoproteins *O*-Glycans	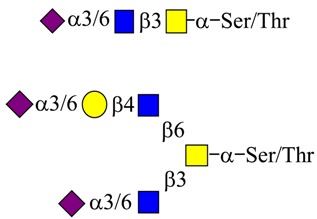 Mucin type *O*-Glycans Linear sialylated core 3 Branched sialylated core 4
Glycoproteins *O*-GlcNAcylation	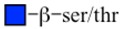
Glycoproteins *C*-Mannose	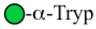

The main linkages of glycans to proteins are listed.

**Table 5:** Key Glycan Structures found in Mucins.

Type of Glycan	Structure
Blood group H type 1 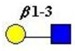 (Galβ1-3GlcNAc)	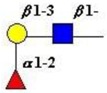 Fucα1-2Galβ1-3GlcNAcβ1-
Blood group A type 2 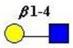 (Galβ1-4GlcNAc)	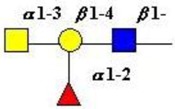 GalNAcα1-3Galβ1-4GlcNAcβ1- | α1-2 Fuc
Blood group B type 1 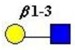 (Galβ1-3GlcNAc)	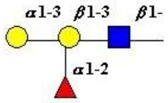 Galα1-3Galβ1-3GlcNAcβ1- | α1-2 Fuc
Lewis^a^ and Lewis^x^	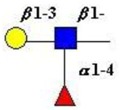 Galα1-3GlcNAcβ1- | α1-4 Fuc 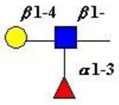 Galβ1-4GlcNAcβ1- | α1-3 Fuc
Lewis^b^	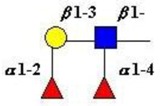 Galβ1-3GlcNAcβ1- | α1-2 | α1-4 Fuc Fuc
Lewis^y^	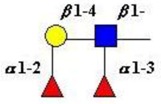 Galβ1-4GlcNAcβ1- | α1-2 | α1-3 Fuc Fuc
Sialyl Lewis^a^ and Sialyl Lewis^x^	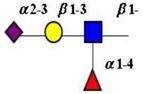 Neu5Acα2-3Galβ1-3GlcNAcβ1- | α1-4 Fuc 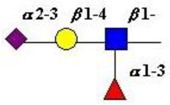 Neu5Acα2-3Galβ1-4GlcNAcβ1- | α1-3 Fuc
Sialyl-Tn	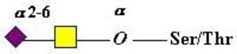 Neu5Acα2-6GalNAc-α-*O*-Ser/Thr
Monosialylated-T-antigen	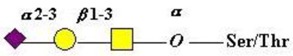 Neu5Acα2-3Galβ1-3GalNAc-α-*O*-Ser/Thr 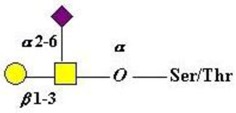 Neu5Ac | α2-6 Galβ1-3GalNAc-α-*O*-Ser/Thr
Monosialylated core 3	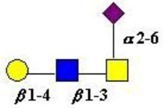 Neu5Ac | α2-6 Galβ1-4GlcNAcβ1-3 GalNAc
Sd^a^ antigen Type 1 & 2 chains	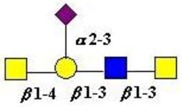 Neu5Ac | α2-3 GalNAcβ1-4Galβ1-3GlcNAcαβ1-3GalNAc 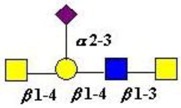 Neu5Ac | α2-3 GalNAcβ1-4Galβ1-4GlcNAcαβ1-3GalNAc-

Some examples of important glycan structures commonly found in mucin glycans are shown.

**Table 6:** Nucleotide Forms for Transfer to Mucins.

Transfer Moiety	Nucleotide	Nucleotide Transport ER	Nucleotide Transport Golgi	Comment
Neu5Ac	CMP-Neu5Ac	−	+	Golgi location. Also transfers Neu5Gc
Fuc	GDP-Fuc	+	+	ER and Golgi location
Gal	UDP-Gal	−	+	Only in Golgi
Man	GDP-Man	−	+	Only in Golgi
GlcNAc	UDP-GlcNAc	+	+	ER and Golgi location
GalNAc	UDP-GalNAc	+	+	ER and Golgi location
Sulphate	PAPS *	−	+	Only in Golgi
Acetate	Acetyl-CoA	+	+	ER and Golgi location
Acyl	Acyl-CoA	?	?	Not known
Methyl	S-adenosyl-methionine	?	?	Not known
Phosphate	ATP	+	+	ER and Golgi location

The Table shows the nucleotide monosaccharide forms active as substrates for the glycosyltransferases. In addition the donors for transfer of sulfate, acetate, acyl, methyl, and phosphate groups found in the glycosylation pathways mucin is listed. The location of the nucleotide transfer is indicated where known. Detail of the monosaccharide metabolic pathways is shown in Figure 4. * PAPS, 3′-phosphoadenosine-5′-phosphosulphate.

**Table 7:** Expression of Mucin Gene Proteins and Mucin Glycosylation in Diverted and Non-Diverted Colonic Tissue.

Structure	Reagent	Reference	Non-Diverted *n* = 50	Diverted *n* = 35	*p*
***Mucins***					
MUC1	HMFG2	[345]	strong	no change	NS
MUC2	LUM2-3	[346]	strong	no change	NS
MUC3A/B	EU MUC3	European Union consortium	weak	no change	NS
MUC4	M4.275	[347]	strong	no change	NS
MUC5AC	21M1	[348]	negative	no change	NS
MUC5B	LUM5B-2	[349]	weak	no change	NS
MUC12	M11.123	[350]	strong	no change	NS
MUC13	M13.234	[351]	strong	no change	NS
***Glycosylation***					
**Antibodies**					
Tn	IE3	[352]	weak	no change	NS
Sialyl-Tn	TKH2	[353]	weak	strong	<0.001
Sialyl-Le^a^	CA19.9	[354]	weak	strong	<0.01
Sialyl-Le^x^	AM3	[355]	strong	strong	NS
Sulpho-Le^a^	91.9H	[356]	strong	strong	NS
Sulpho-Le^a^	F2	[357]	strong	strong	NS
Sd^a^	KM694	Kyowa Hakko Kogyo Co. Ltd., Tokyo, Japan	strong	negative	<0.001
*O*-Acetyl sialic acid 1	PR3A5	[358]	strong	strong	NS
*O*-Acetyl sialic acid 2	6G4	[359]	strong	negative	<0.001
**Lectins**					
Sialyl-α2-3	MALII	[360]	strong	strong	NS
Sialyl-α2-6	SNA	[361]	strong	strong	NS
GalNAc-protein	VVA	[362]	strong	strong	NS
Fucosyl-α1-2	UEA1	[363]	weak	weak	NS
Fucosyl-α1-6	AAA	[363]	strong	strong	NS
Galβ1-3GalNAc	PNA	[364]	strong	strong	NS
Galβ1-4 GlcNAc	MALI	[365]	strong	strong	NS
GalNAcα1-3GalAc	DBA	[366]	strong	negative	<0.001
β1-3Gal					
GalNAcβ1-4					
(Neu5Acα2-3)Gal		[367]			

The LUM2-3, LUM5B-2, and EU MUC3 antibodies were raised in the European Union consortium (grant CEEBMH4- CT98-3222). The KM694 antibody was a gift from the Kyowa Hakko Kogyo Co. Ltd., Tokyo, Japan. M4.275, M11.123, and M13.234 antibodies were provided by Dr. M.A. McGuckin, University of Queensland, Brisbane, Australia. The F2 antibody was provided by Dr. E.C.I. Veerman and Dr. A.V. Nieuw Amerongen, Vrije University, Amsterdam, The Netherlands. IE3 antibody was a gift from Prof, S. Hakomori, Univ Seattle, USA. Prof T. Irimura, University of Tokyo, Japan provided the CA19.9 antibody. AM3 antibody was a gift from Prof C. Hanski, University of Berlin, Germany. All lectins were obtained from Vector Laboratories, Peterborough, UK. Statistical analysis was performed using Unistat software. Non-parametric data were compared using the Mann–Whitney *U*-test and matched pairs were compared using the Wilcoxon Signed Rank test. *p* values are shown and *p* > 0.05 was taken as non-significant (NS). Abbreviations for the reagents are given at the end of the paper.

**Table 8:** Sialic acid *O*-acetylation in IBD.

Patient Group	mPAS			PR3A5			6G4		
	Total	Positive	Non-Acetylator	Total	Positive	*p*	Total	Positive	*p*
UC	30	25	5	30	25		25		
UC div	17	14	3	17	14	>0.05	14		0.0003
CD	9	19	0	9	8		9		
CD div	17	15	2	17	15	>0.05	17		0.0112
Non IBD	19	16	3	19	16		9	13	
Non IBD div	15	15	0	15	15	>0.05	15	5	0.0113

The Table shows diverted and non-diverted patients with UC, CD, or non-IBD colonic disease. The mPAS data was carried out with and without saponification and also indicates the non-*O*-acetylators in the disease groups. The mPAS data showed no significant differences between the diverted/non-diverted groups. The *p* values are for diverted vs. non-diverted patients.

## Abbreviations Used in the Text Tables and Figures

**Monosaccharides:** Glc, d-glucose; Gal, d-galactose; Man, d-mannose; GlcN, d-glucosamine; GalN, d-galactosamine; ManN, d-mannosamine; GlcNAc, *N*-acetyl-d-glucosamine; GalNAc, *N*-acetyl-d-galactosamine; ManNAc, *N*-acetyl-d-mannosamine; Fuc, l-fucose; Neu5Ac, *N*-acetylneuraminic acid; Neu5Gc, *N*-glycoylneuraminic acid; P, phosphate; S, sulphate.

**Lectins:** MALI, Maackia amurensis lectin I; MALII, Maackia amurensis lectin II; SNA, Sambucus nigra agglutinin; VVA, Vicia villosa agglutinin; UEA1, Ulex europaeus agglutinin1; AAA, Anguilla anguilla agglutinin; PNA, peanut, Arachis hypogaea agglutinin; DBA, Dolichos biflorus agglutinin.

## Symbols for Individual Monosaccharides Used in Figures

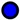
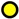
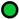
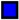

			
d-glucose;	d-galactose;	d-mannose;	*N*-acetyl-d-glucosamine;

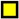
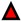
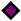

		
*N*-acetyl-d-galactosamine;	l-fucose;	*N*-acetylneuraminic acid.

Symbols are as recommended [521].
